# Sculpting Rupture‐Free Nuclear Shapes in Fibrous Environments

**DOI:** 10.1002/advs.202203011

**Published:** 2022-07-21

**Authors:** Aniket Jana, Avery Tran, Amritpal Gill, Alexander Kiepas, Rakesh K. Kapania, Konstantinos Konstantopoulos, Amrinder S. Nain

**Affiliations:** ^1^ Department of Mechanical Engineering Virginia Tech Blacksburg VA 24061 USA; ^2^ Department of Chemical and Biomolecular Engineering Johns Hopkins University Baltimore MD 21218 USA; ^3^ Kevin T. Crofton Department of Aerospace Engineering Virginia Tech Blacksburg VA 24061 USA

**Keywords:** cell forces, cell spreading, cell‐cytoskeleton, extracellular matrices, nanofibers, nucleus shapes, yes‐associated protein/TAZ

## Abstract

Cytoskeleton‐mediated force transmission regulates nucleus morphology. How nuclei shaping occurs in fibrous in vivo environments remains poorly understood. Here suspended nanofiber networks of precisely tunable (nm–µm) diameters are used to quantify nucleus plasticity in fibrous environments mimicking the natural extracellular matrix. Contrary to the apical cap over the nucleus in cells on 2‐dimensional surfaces, the cytoskeleton of cells on fibers displays a uniform actin network caging the nucleus. The role of contractility‐driven caging in sculpting nuclear shapes is investigated as cells spread on aligned single fibers, doublets, and multiple fibers of varying diameters. Cell contractility increases with fiber diameter due to increased focal adhesion clustering and density of actin stress fibers, which correlates with increased mechanosensitive transcription factor Yes‐associated protein (YAP) translocation to the nucleus. Unexpectedly, large‐ and small‐diameter fiber combinations lead to teardrop‐shaped nuclei due to stress fiber anisotropy across the cell. As cells spread on fibers, diameter‐dependent nuclear envelope invaginations that run the nucleus's length are formed at fiber contact sites. The sharpest invaginations enriched with heterochromatin clustering and sites of DNA repair are insufficient to trigger nucleus rupture. Overall, the authors quantitate the previously unknown sculpting and adaptability of nuclei to fibrous environments with pathophysiological implications.

## Graphical abstract

Altered apicobasal polarity in ECM‐mimicking fibrous environments sculpts rupture‐free nuclei through fiber curvature‐driven contractility. Cells on single fibers have deep nucleus invaginations at fiber‐specific sites causing preferential enrichment of heterochromatin and DNA damage. Force‐driven nuclear compression in cells on multiple fibers correlates with enhanced YAP nuclear translocation. Mismatch‐diameter combinations lead to teardrop‐shaped nuclei due to anisotropy in actin stress‐fiber distribution.

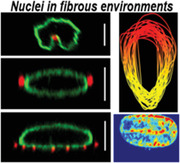

## 1 Introduction

The nucleus, as the stiffest cellular organelle, plays a central role in mechanotransduction.^[^ ^1^, ^2^ ^]^ Proper control of nuclear shapes is one of the primary cellular functions controlling cell migration, differentiation, and tissue morphogenesis.^[^ ^3^ ^]^ Thus, not surprisingly, defects in nuclear shapes are often implicated in various disease states, including progeria, muscular dystrophy, and cancer metastasis.^[^ ^4^ ^]^ Mechanical forces regulate nuclear shapes^[^ ^5^, ^6^, ^7^ ^]^ originating from cells' dynamic interactions with their surrounding microenvironment, that is, the extracellular matrix (ECM), and transmitted to the nucleus by the cell cytoskeleton. The nuclear envelope composed of the lamin intermediate filament networks is critical in sustaining external forces exerted on the nucleus. Lamin network mutations and deficiencies cause significant reduction of the nuclear stiffness,^[^ ^8^ ^]^ leading to nuclear blebbing,^[^ ^9^ ^]^ and mislocalization of DNA repair factors, and DNA damage.^[^ ^10^ ^]^

Mechanical forces primarily act through the actomyosin contractions in the stress fibers to control nucleus shape to induce nucleo‐cytoplasmic shuttling of transcription factors like yes‐associated protein [YAP], transcriptional coactivator with PDZ‐binding motif (TAZ), megakaryoblastic leukemia 1 (MKL1), and chromatin remodeling proteins such as histone deacetylases (HDAC).^[^ ^11^, ^12^ ^]^ The force‐mediated nuclear shape changes also induce reorganization and stretching of the internal chromatin domains,^[^ ^13^ ^]^ resulting in altered transcriptional activity and mechanical properties of the nucleus.^[^ ^14^ ^]^ A growing consensus in the field has been that nuclei rupture as they undergo drastic shape changes, causing mislocalization of signaling molecules (6, 12). Large scale mechanical forces, driven by ECM stiffness, mold cell shape, which controls the nucleus shape; high aspect ratio nuclei induce high curvature at the poles, making them susceptible to rupture.^[^ ^9^ ^]^

In vivo 3D aligned matrices, frequently observed in healthy and diseased states, including tendons,^[^ ^16^, ^17^ ^]^ muscle tissue,^[^ ^18^, ^19^ ^]^ and extracellular regions surrounding metastatic tumors,^[^ ^20^, ^21^, ^22^ ^]^ provide topographic cues for cells to spread uniaxially and migrate persistently by exerting forces.^[^ ^23^, ^24^ ^]^ Fibrillar matrices composed of individual collagen fibrils range in size from 70 to 300 nm^[^ ^25^, ^26^, ^27^, ^28^ ^]^ that can bundle to larger fibers varying from 1 to 20 µm in diameter.^[^ ^26^, ^28^ ^]^ Mimicking the native ECM architecture within an in vitro setting is often highly challenging, and flat 2D substrates with and without anisotropic features and 3D gels have been used extensively to study cell behavior.^[^ ^29^ ^]^ 2D systems have limited physiological relevance. While 3D collagen gels capture the fibrillar architecture, the inherent heterogeneity in these matrices renders it difficult to understand the role of fiber dimensions and organization in regulating cytoskeletal and nuclear responses.^[^ ^30^, ^31^ ^]^ Studies by the Yamada group have demonstrated how single‐cell behavior in 3D microenvironments can be recapitulated through the use of narrow 1D microprinted lines of varying widths (1–40 µm).^[^ ^32^ ^]^ Microprinted lines are essentially 2D surfaces, and in our study, we inquired if suspended 1D fibrillar architecture of precisely controlled fiber diameters (150–6000 nm) and inter‐fiber spacing regulated nuclear responses of uniaxial spread cells, as we have previously shown protrusive, contractile, and migratory behavior of uniaxial cells to be sensitive to fiber curvature and spacing.^[^ ^33^, ^34^, ^35^, ^36^, ^37^, ^38^, ^39^ ^]^ In this study, we chose nucleus rupture and the spatial localization of YAP/TAZ as two markers of nuclear response to changes in fiber curvatures. We discovered that cytoskeletal and lamin networks in suspended cells are localized in an almost uniform caging structure surrounding the nucleus, contrary to the preferential apical localization in flat continuous surfaces. Fiber‐curvature driven cytoskeleton tension led to precise sculpting of nucleus shape, including unique teardrop shapes due to actin stress fiber anisotropy and invaginations that ran the length of the nucleus. We found that all nucleus shapes, including the sharpest invaginations formed on nanofibers, did not undergo rupture events, indicating remarkable adaptability of nuclei to fibrillar environments. Nuclear translocation of YAP increased with the diameter or fiber density, while nucleus invaginations sites had increased number of heterochromatin clusters. Overall, we describe cellular mechanosensitivity unique to fiber matrices with implications in pathophysiology.

## 2 Results

### 2.1 Suspended Cells Show Uniform Nuclear Caging of Cytoskeletal Elements and Lamins

**Figure 1:**
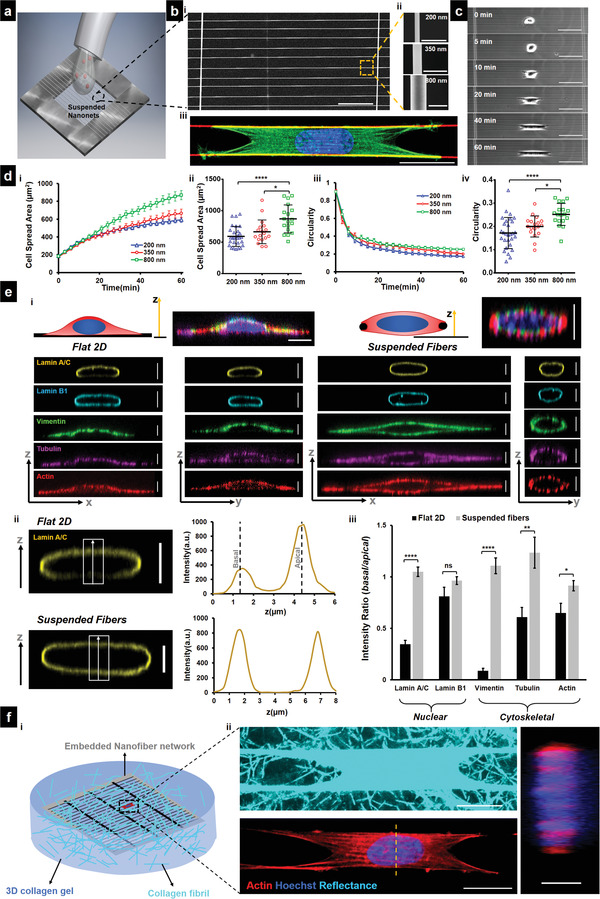
Cell spreading on suspended fiber networks: a) Schematic of assay used for studying cell spreading. Cell suspension is dropped on top of suspended nanonets coated with fibronectin and imaged in real‐time. b[i]) SEM image of suspended nanonet (Scale bar = 50 µm). b[ii]) SEM images of three different fiber diameters 200, 350, and 800 nm used for the study, (Scale bar = 1 µm) and b[iii]) representative cell (stained for Actin‐green, DAPI – blue, and fiber – red) on a 2‐fiber (diameter = 350 nm) doublet (Scale bar = 20 µm). c) Images from time lapse microscopy showing different phases of cell spreading on a 2‐fiber doublet. (Scale bar = 50 µm) d[i,iii]) Temporal evolution of cell spreading area and circularity, respectively (*n* = 16–29 cells per category). d[ii,iv]) Statistical comparison of cell spread area and circularity, respectively, after 1 h of spreading. e[i]) Representative immunofluorescence images showing differences in localization of cytoskeletal elements and nuclear envelope proteins between Flat 2D and suspended nanonets. Cytoskeletal elements form a “caging” structure surrounding the nucleus, while on 2D they “cap” the nucleus from apical side (Scale bars = 5 µm). e[ii]) Representative confocal side views (*xz*) along with intensity profiles along the *z*‐direction, first and second intensity peaks correspond to basal and apical surfaces, respectively (Scale bar = 5 µm). e[iii]) Comparison of the basal and apical intensity for the different cytoskeletal and nuclear envelope proteins (*n* = 10–11 per category) demonstrating intensity ratio values close to 1 for suspended nanonets (diameter = 200 nm) due to caging of the nucleus, absent in cells on 2D. Images and data are shown for C2C12 myoblasts: f[i]) Schematic showing cells attached to a nanofiber network embedded in a 3D collagen gel with an image of a representative embedded cell stained for actin and nucleus (Scale bar = 20 µm). [ii] Collagen gel fibrils are visualized via confocal reflectance microscopy, and cross‐sectional views demonstrate nuclear caging by actin stress fibers (Scale bar = 5 µm). 1‐way ANOVA with Tukey's multiple comparison test was used for (d[ii,iv]), Student's *t*‐test was used for comparison between Flat 2D and suspended fibers for each cytoskeletal and nuclear envelope protein in (e[iii]), *,**,***,**** represent *p* < 0.05, 0.01, 0.001, and 0.0001, respectively.

We wished to compare the responses of cells plated on flat 2D as opposed to those attached to suspended fibers of varying diameters. We utilized force‐measuring nanonets composed of large diameter (≈2000 nm) “strut” fibers fused to orthogonal small diameter aligned fibers (Nanonet force microscopy, NFM).^[^ ^38^, ^40^, ^41^, ^42^ ^]^ To investigate the role of fiber diameter, we selected three different fiber diameters: 200, 350, and 800 nm, while keeping the spacing between large strut fibers constant (≈250 µm). Fiber beam stiffness, midspan of a beam scales approximately with ≈*d* ^2^; thus, constant beam length allows us to directly compare the effects of fiber diameter contributing to an increase in stiffness.^[^ ^33^, ^38^, ^40^ ^]^ We investigated the dynamics of cell spreading and the organization of cytoskeletal networks using a custom setup to add cell suspension droplets on the fibronectin‐coated fiber networks (**Figure** **1**a,b). The inter‐fiber spacing (10–12 µm) was chosen to ensure that cells spread along 2 fibers to form symmetric parallel‐cuboidal shapes (Figure 1b[iii]). We monitored cell spreading using optical microscopy for cells attached approximately midway across the fiber span length (Figure 1c). After making initial contact with the fiber networks, cells began to protrude along the nanofibers (Movie S1, Supporting Information). Consistent with our previous findings,^[^ ^34^ ^]^ we observed protrusions formed during spreading were primarily actin‐based, while microtubules and vimentin intermediate filaments localized later during the spreading cycle (Figure S1b, Supporting Information). To characterize cell size and shape during spreading, we tracked the cell projected area and the circularity for 1 h for the three diameters (Movies S2–S4, Supporting Information). The cell area growth curves for all the fiber diameter categories showed a faster initial incremental phase followed by gradual maturation at longer times (Figure 1d[i,iii]), matching growth rate kinetics on 2D substrates.^[^ ^43^ ^]^ Interestingly, we found that cells achieved steady‐state area and circularity faster (≈40 min) on 200 nm diameter fibers, while cells on larger diameter fibers demonstrated higher spread areas and circularity (Figure 1d[ii,iv]).

Next, we inquired if classic apical localization of cytoskeletal elements, described in studies on flat surfaces, extended to cells attached to suspended nanonets. On 2D substrates, nuclear shapes are shown to be regulated by cell boundary movements that involve both the actomyosin cytoskeleton's contractility and the mechanical tension of the cell membrane.^[^ ^44^, ^45^ ^]^ Khatau et al. identified the apical perinuclear actin cap as a primary regulator of the nuclear shape.^[^ ^46^ ^]^ In contrast, more recently, Ihalainen et al. demonstrated a differential localization^[^ ^47^ ^]^ of the nuclear lamins (particularly Lamin A/C) toward the apical nuclear envelope. Informed by these studies, we examined the localization of the major cytoskeletal elements (f‐actin stress fibers, microtubules, and vimentin intermediate filaments) and nuclear lamins (A/C and B1) in cells attached to flat glass surfaces and on our suspended nanonets. We observed that the f‐actin, microtubule, and intermediate filament cytoskeleton was highly aligned along the fiber axis in elongated cells compared to their counterparts on flat glass (Figure S1a,c, Supporting Information). Confocal microscopy side‐views (*xz* and *yz*) at various stages during cell spreading revealed significant differences in the localization of the nuclear lamins and the overall organization of the cytoskeletal elements around the nucleus (Figure 1e[i] and Figure S2, Supporting Information). On flat 2D, our findings on apical‐basal localization of Lamin A/C were in agreement with previously reported literature (Intensity ratio_basal/apical_ = 0.35 ± 0.04), but contrasted on suspended nanonets (Intensity ratio_basal/apical_ = 1.05 ± 0.05). In a similar manner, cells on flat glass displayed preferential enrichment of different cytoskeletal elements toward the apical side in a “capping” manner (Figure S2a, Supporting Information), with the strongest apical preference observed in the case of the vimentin intermediate filaments (Intensity ratio_basal/apical_ = 0.09 ± 0.02). Contrarily, the cytoskeletal network was nearly uniformly distributed around the nucleus in a “caging” manner on suspended nanonets (intensity ratios ≈ 1, Figure 1e[iii]).

To investigate if the cytoskeletal caging observed here, also extended to cells inside 3D adhesive spaces, we cultured cells on our ECM‐mimicking fiber networks embedded within 3D collagen gels (Figure 1f[i]). Cells embedded within such gels still demonstrated cytoskeletal caging of the nucleus (Figure 1f[ii] and Figure S3, Supporting Information).

Overall, we conclude that cytoskeletal caging of the nucleus in 3D microenvironments is universal and such caging is the major contributor toward the uniform distribution of nuclear lamins, which is distinctly different than observations from 2D substrates.

### 2.2 Increase in Cytoskeletal Tension during Cell Spreading Is Fiber‐Diameter Dependent

**Figure 2:**
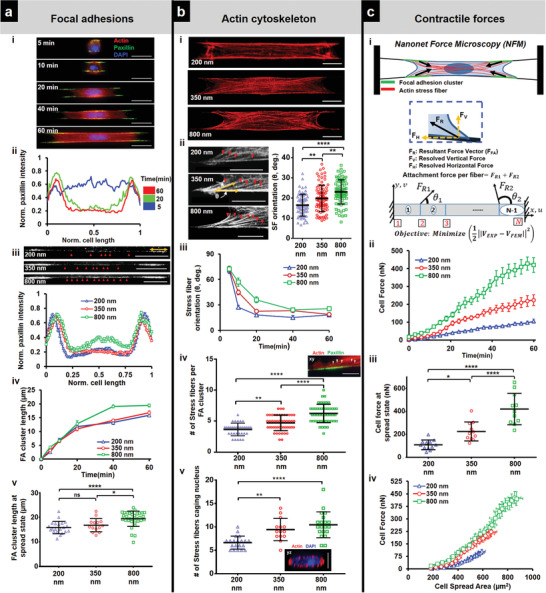
Cell contractility during spreading: a) Focal adhesion (FA) organization: a[i]) Representative images showing cells stained for actin (red), paxillin (green), and DAPI (blue) at different timepoints during spreading (fiber diameter = 350 nm) (Scale bars = 20 µm). a[ii]) Normalized paxillin intensity (average of 9–12 profiles for each timepoint) showing spatiotemporal reorganization of focal adhesions as cells spread, a[iii]) Differences in FA organization in clusters (yellow arrow) at the poles and along cell body‐fiber length (red arrowheads) for different fiber diameters (Scale bars = 20 µm). a[iv]) Temporal evolution of FA cluster lengths. a[v]) Comparison of FA cluster lengths at final spread state, *n* = 21, 14, and 33. b) Actin cytoskeleton organization: b[i]) Maximum intensity projections of actin cytoskeleton at spread state (60 min) (Scale bars = 10 µm). b[ii]) Stress fiber angle formed at the focal adhesion cluster zones, *n *= 68, 88 and 80 (Scale bars = 10 µm). b[iii]) Transient evolution of orientation of the stress fibers during spreading. b[iv]) Number of major stress fibers originating from each FA clustering zone, *n* = 33, 49, and 47. b[v]) Number of major stress fibers caging the nucleus, *n* = 20, 14, and 20. Inset are representative images of FA clustering zone (Scale bars = 10 µm) and nucleus caging with actin networks (Scale bars = 5 µm). c) Contractile forces: c[i]) Schematic of force measurement using Nanonet Force Microscopy (NFM) technique. c[ii]) Transient cell attachment force evolution during spreading (*n* = 11–12 cells each diameter category). c[iii]) Comparison of cell forces at spread state between the different fiber diameters. c[iv]) Relationship between cell forces and cell spread area. Cell forces are computed from both fibers. Sample sizes are given for 200–200, 350–350, and 800–800 nm diameter doublets, respectively. Images and data are shown for C2C12 myoblasts.

Since cells were achieving steady‐state spreading fastest on the smallest diameters and suspended networks were causing cytoskeletal elements to cage the nucleus, we inquired about the role of focal adhesions and actin networks in establishing contractile forces. Visualizing cell–fiber adhesions through paxillin immunostaining at various time points (5, 10, 20, 40, and 60 min) during cell spreading revealed that focal adhesion (FA) clusters were formed along the entire cell length at early stages (*t* ≈ 10 min). In contrast, a preferential localization of the FA clusters occurred to the cell poles with increased spreading (**Figure** **2**a[i] and Figure S4, Supporting Information). Normalized paxillin intensity taken along the cell length revealed two distinct peaks (Figure 2a[ii]) corresponding to the major FA clusters at either cell pole, consistent with our previous findings.^[^ ^33^, ^38^, ^39^ ^]^ The transition of adhesion sites from being punctate along the entire cell body to localizing in major clusters at cell poles occurred by the 20‐min time point (Figure 2a[ii]). A closer inspection of the adhesion distribution along the cell length at 60‐min (Figure 2a[iii]) revealed smaller paxillin clusters distributed along the cell–fiber length, which became more prominent on larger diameter (800 nm) fibers (red arrowheads). FA cluster lengths were observed to grow in length with cell spread, with the largest 800 nm diameter fibers resulting in the longest cluster lengths at 60‐min time point (Figure 2a[iv,v]).

Our observations that FA clustering occurred in a fiber diameter‐dependent manner suggested that fiber diameter might also influence the associated cytoskeletal tension. We immunostained for the contractile f‐actin cytoskeleton (Figure 2b[i]) in cells at various time points (5, 10, 20, 40, and 60 min) and found that the average angle made by stress fibers anchored to the FA clustering sites at poles progressively decreased as cells spread, with the shallowest angle formed on 200 nm diameter fibers (Figure 2b[ii,iii]). We observed that the number of stress fibers originating at each FA cluster zone increased with fiber diameter, indicating increased contractility (Figure 2b[iv]). We also investigated the actin cytoskeletal organization in the perinuclear region since the actin stress fibers in this region directly affect nucleus shape regulation. Appearing as individual dots surrounding the nucleus, in confocal cross sections (*yz*, Figure 2b[v]), we found that the number of stress fibers originating from individual FA clustering regions were less than those in the perinuclear region due to convergence of stress fibers emanating from FA clusters on either side, a behavior unique to anisotropically stretched cells in suspended nanonets (Figure S5, Supporting Information).

Using NFM, we estimated the forces in cell spreading by monitoring the contractile inward deflection of fibers (Figures 1c and 2c[i]). Fiber deflections were subsequently converted into attachment forces at the individual FA clusters using inverse methods that minimize the error between computed and experimentally observed fiber deflections (Experimental Section). The inputs to the computational framework include fiber properties and force vectors that originate from FA clustering zones (Figure 2a[iii]) and are directed along the average stress fiber orientation per fiber category (Figure 2b[ii,iii]). Consistent with our finding that the number of stress fibers increases with fiber diameter, the computed forces (*F*_cell/fiber_ = *F*_R1_ + *F*_R2_) also increase as cells spread and with an increase in diameter (Figure 2c[ii,iii]). We also plotted cell force against the cell spread area (Figure 2c[iv]). We found that for the same area across different diameters, cells attached to larger diameter fibers exerted significantly higher forces. Overall, our data suggest that increasing the fiber diameter causes cells to form larger focal adhesion clusters at the poles, leading to an increased number of actin stress fibers resulting in higher cell contractility.

### 2.3 Nuclear Translocation of Transcription Factors Is Regulated by Nuclear Compression

**Figure 3:**
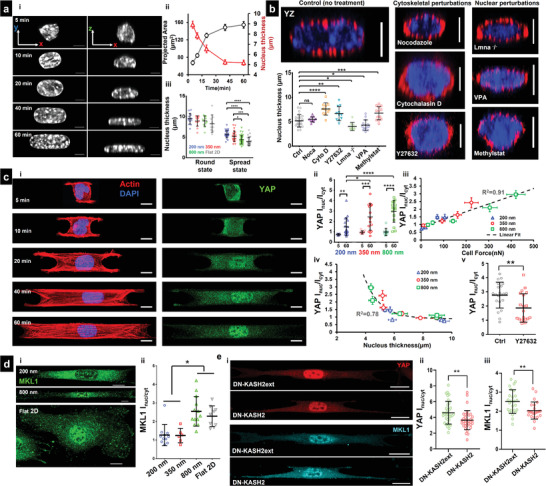
Nuclear compression and YAP localization during spreading: a[i]) Representative images showing top view (*xy*) and side view (*xz*) of nuclei at different timepoints during cell spreading (fiber diameter = 350 nm), (Scale bars = 5 µm). a[ii]) Increase in nucleus projected area (*xy*) and decrease in nucleus thickness (*z*), indicating nucleus flattening with cell spreading, *n* = 13–32 per timepoint. a[iii]) Nucleus thickness comparison between rounded (5 min) and spread (60 min) state, *n* = 14, 13, 12, and 9 (for round state) and *n* = 32, 25, 36, and 22 (for spread state) for 200, 350, 800 nm, and Flat 2D, respectively. b) Representative cross‐sectional (*yz*) images of cells under various drug conditions and lamin A/C knockdown, *n* = 25, 10, 11, 11, 9, 14, and 13 (Scale bars = 5 µm), Comparison of nucleus thickness with control (no treatment), fiber diameter = 350 nm. c) Nuclear entry of YAP during cell spreading: c[i]) Representative images at various timepoints during spreading (Scale bars = 10 µm). c[ii]) Comparison of nuclear/cytoplasmic YAP between rounded (5 min) and spread state (60 min) for different fiber diameters, *n* = 10–15 cells per timepoint and diameter category. c[iii]) Increase in nuclear YAP correlates with cell forces. c[iv]) Increase in nuclear YAP correlates with nucleus thickness. c[v]) Nuclear YAP localization decreases with loss of contractility through pharmacological inhibition, *n* = 21 and 20 for control and Y27632 treatment. Fiber diameter = 800 nm). d[i]) Representative cells showing MKL1 on 200 and 800 nm fiber doublets and on Flat 2D substrate (Scale bars = 10 µm). d[ii]) Quantification of the nuclear/cytoplasmic MKL1 on 200, 350 and 800 nm fiber doublets and Flat 2D. e[i]) Representative cells showing YAP and MKL1 localization on DN‐KASH2ext and DN‐KASH2 cells (Scale bars = 20 µm). e[ii,iii]) Quantification of the nuclear/cytoplasmic YAP and MKL1, respectively, with DN‐KASH2ext and DN‐KASH2 cells, Fiber diameter = 800 nm. Images and data are shown for C2C12 myoblasts.

Given our observations on the arrangement of F‐actin networks caging the nucleus, we sought to investigate how the compression forces impacted the nucleus geometry and translocation of various transcription factors, including YAP,^[^ ^48^ ^]^ known for its central role in mechanotransduction. To determine nucleus geometry, we used confocal microscopy on DAPI‐stained cells (**Figure** **3**a[i]) that were fixed at various time points (5, 10, 20, 40, and 60 min). We employed three parameters: nucleus projected area (size in *xy* plane, top view), nucleus eccentricity (shape in *xy* plane), and the nucleus thickness (compression in *xz* plane, side view). Nucleus projected area increased steadily and reached an equilibrium value for all the fiber diameters tested (Figure 3a[ii] and Figure S6a, Supporting Information), with cells in 200 nm nanonets reaching stable areas the fastest. As cells spread, we observed the nuclei elongated (rise in eccentricity, Figure S6b, Supporting Information). Not surprisingly, as the nuclei underwent compression from the near‐spherical shape in rounded cells to the flattened “pancake” shape (confocal side views, Figure 3a[i]), the nucleus thickness reduced significantly over time (Figure 3a[ii]), with the minimum thickness in cells attached to 800 nm nanonets (Figure 3a[iii]), suggesting that the cytoskeletal tension primarily drove the nuclear compression.

To test the relative contributions of the actin and microtubule cytoskeletons in regulating force‐driven nuclear compression, we treated cells on nanonets with either cytochalasin D (2 µm) or nocodazole (1 µm). As expected, cytochalasin D treatment led to cells of reduced spread area (Figure S7, Supporting Information) and disrupted actin cytoskeleton as evidenced by loss of stress fibers caging the nucleus (Figure 3b). Under these conditions, the nucleus thickness was significantly (≈1.5×) higher than the control cells (no drugs). In contrast, disruption of the microtubule cytoskeleton did not significantly alter the nuclear compression levels. Next, we reduced cytoskeletal tension and actin stress fiber formation through the selective ROCK inhibitor Y27632 and observed nuclei of significantly larger thickness (Figure 3b), further elucidating the role of the actin cytoskeleton caging network in nucleus shape maintenance.

Informed by these findings, next, we investigated the role of the nuclear lamina in sustaining cytoskeletal forces. The nuclear lamina, composed of the nuclear lamins (A, C, B1, and B2), is a thin intermediate filament meshwork enveloping the nucleus, providing essential mechanical support. We generated C2C12 lamin A/C KD cells with wrinkled nucleus morphologies (Figure S8, Supporting Information) consistent with observations from previous studies.^[^ ^49^ ^]^ Fully spread Lamin KD cells had lower nucleus thickness than control, likely due to decreased nuclear stiffness.^[^ ^15^ ^]^ Next, we altered the chromatin compaction levels by pre‐treating cells with the histone deacetylase inhibitor (HDACi) valproic acid (VPA) used for chromatin decompaction and reducing nucleus stiffness or the histone demethylase inhibitor, Methylstat, used for chromatin compaction and increasing nuclear stiffness.^[^ ^8^, ^50^ ^]^ Cells treated with VPA or Methylstat demonstrated significantly decreased and increased nucleus thickness, respectively, compared to control cells (Figure 3b).

Recent studies have shown how cytoskeletal‐mediated compressive forces acting on the nucleus can stretch the nuclear membrane pores.^[^ ^12^ ^]^ We wanted to inquire if the nuclear entry of YAP correlated with the fiber‐diameter driven nuclear compression. Immunostaining for YAP (Figure 3c[i]) at various time points during cell spreading showed that YAP localization was primarily cytoplasmic (YAP intensity ratio between nucleus and cytoplasm *I*_nuc_/*I*_cyt_ < 1) during early stages of cell spreading (5–10 min) but with an increase in cytoskeletal tension at later stages of cell spreading, a significant increase in the nuclear entry of YAP was observed (Figure 3c[ii]). Interestingly, we observed that the nuclear YAP translocation ratio was independent of cell shape but dependent upon force and nucleus thickness (Figure 3c[iii,iv]). Thus, reducing nuclear compression through contractility inhibition (Y27632 treatment) resulted in a significant decrease in the nuclear translocation of YAP (Figure 3c[v]).

We extended our interrogations to include nucleo‐cytoplasmic shuttling of other known mechanosensitive transcription factor MKL1, which demonstrated a similar increase in nuclear entry in large diameter fiber doublets (Figure 3d[i,ii]).

Next, we inquired how crucial cytoskeletal tension‐mediated nuclear deformation is for nuclear entry of such transcription factors. To this end, we perturbed force transmission from the cytoskeleton to the nucleus by utilizing a dominant‐negative KASH2 (GFP‐DN‐KASH) construct.^[^ ^6^ ^]^ DN‐KASH induced uncoupling of nucleus from the cytoskeleton reduced both YAP and MKL1 nuclear localization by 25%, compared to the control DN‐KASH2ext, which is targeted to the outer membrane but cannot disrupt the LINC complex (Figure 3e[i–iii]). This suggests that fiber‐induced nuclear deformation is sufficient to contribute to nuclear translocation of transcription factors.

### 2.4 Mismatch Diameter Fiber Networks Sculpt Asymmetric Nuclear Shapes and Invaginations

**Figure 4:**
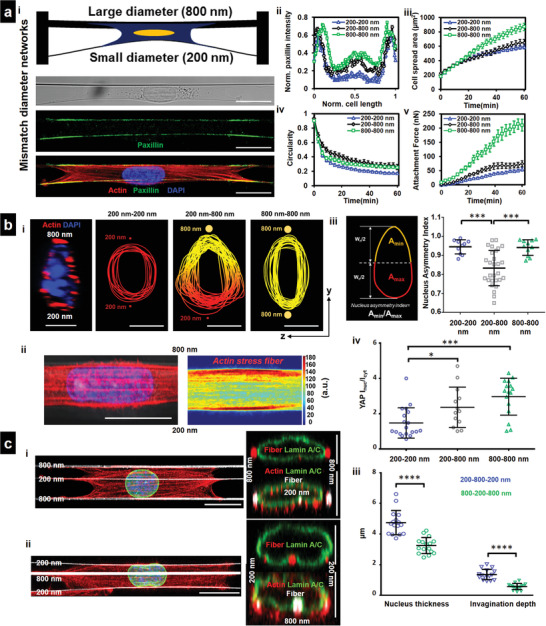
Sculpting nucleus shapes using mismatch diameter networks. a[i]) Cells attached to a small diameter (200 nm) fiber coupled on other side to a large diameter (800 nm) fiber (Scale bars = 20 µm). a[ii]) Mismatch fiber combination leads to enhanced focal adhesion organization along smaller diameter fiber, *n* = 10–14 per category. a[iii]) Increased cell spread area compared to 200–200 nm networks. a[iv]) Altered cell shape, *n* = 12–17 per category. a[v]) Force exertion on 200 nm diameter fiber in 200–800 nm mismatch configuration is higher than in 200–200 nm configuration, *n* = 10–12 per category, b[i]) Mismatch (2‐fiber, 200–800 nm) networks induce asymmetric (teardrop) nucleus shape, nuclear cross sections (*yz*) perpendicular to cell length are used (Scale bars = 5 µm). b[ii]) Teardrop shapes are formed due to higher density of actin stress fibers at the 800 nm diameter side of cell, *n* = 17 cells (Scale bars = 20 µm). b[iii]) Analysis of teardrop shapes using Nucleus Asymmetry Index, *n* = 10, 28, and 11 for 200–200, 200–800, and 800–800 combinations, respectively. b[iv]) nuclear/cytoplasmic YAP in mismatch diameter networks, *n* = 19, 13, and 16 for 200–200, 200–800, and 800–800 combinations, respectively. c[i,ii]) 3‐fiber mismatch (800–200–800 and 200–800–200 nm) can induce “invaginations” in nucleus (*yz*‐cross sections, nuclear envelope stained for lamin A/C, green, fibers, red), second representative cross section for each category, is included to demonstrate cytoskeletal caging, actin (red), Lamin A/C (green), fibers (white), and nucleus (blue), (Scale bars = 20 µm for top views, 5 µm for cross‐sectional views). c[iii]) Comparison of the nucleus thickness and the depth of the invaginations show that 200–800–200 nm triplets have thicker nuclei and larger invaginations due to middle 800 nm diameter fiber compared to 800–200–800 category (*n* = 16 for both categories). Images and data are shown for C2C12 myoblasts.

Cells in the native fibrous ECM can interact simultaneously with fibers of different diameter combinations. Hence, we interrogated force dynamics and YAP localization in mismatch diameter nanonets. To this end, we developed a fiber‐spinning strategy using our non‐electrospinning spinneret‐based tunable engineered parameters (STEP^[^ ^51^, ^52^, ^53^ ^]^) platform to deposit nanonets in two mismatch‐diameter combinations (2‐fiber 200–800 nm doublets, and 3‐fiber triplets (200–800–200 nm, and 800–200–800 nm). We reasoned that cell spreading on 200–800 nm doublets would proceed as described in Figure 1, with cells spreading faster on 200 nm side leading to trapezoidal‐shaped cells with a longer base on 200 nm diameter side. Unexpectedly, we found symmetric spreading on both diameters (**Figure** **4**a and Movie S5, Supporting Information) but enhanced focal adhesion clustering (with respect to the 200 nm–200 nm counterparts) along the cell–fiber interface on the 200 nm fiber side (Figure 4a[i,ii]) that was similar to the focal adhesion clustering on 800 nm nanonets (Figure 2a[iii]). Increased cell adhesion sites on the 200 nm fiber side caused cells to spread more, have higher circularities, and exert larger forces on the 200 nm diameter fiber than their counterparts on 200–200 nm nanonets (Figure 4a[iii–v]).

Intrigued by the altered force exerted by cells on mismatch nanonets, we next inquired if YAP localization inside the nucleus was also affected. First, we investigated the differences in nucleus cell shapes. We unexpectedly discovered asymmetric nuclear shapes (teardrop, confocal side view) with the tapered end toward the 800 nm diameter fiber (Figure 4b[i]). Analyzing the actin stress fiber distribution across the cells attached to mismatch diameter doublets revealed a higher density near the larger diameter fibers, potentially being the cause of increased nucleus compression (Figure 4b[ii]). To quantify the teardrop shapes, we defined the nucleus asymmetry index (NAI, Figure 4b[iii], Experimental Section) as *A*_min_/*A*_max_, where *A*_max_ and *A*_min_ are the areas of the larger and smaller regions with respect to the nucleus mid‐width line (Figure 4b[iii]), respectively. Cells adhering to mismatch doublets demonstrated significant asymmetry (NAI ≈ 0.8, Figure 4b[iii]), compared with symmetric nuclei on 200–200 and 800–800 nm nanonets (NAI ≈ 0.95). Furthermore, the nuclear localization of YAP was significantly enhanced in mismatch diameter fiber networks, as compared to the 200–200 nm, but less than 800–800 nm nanonets (Figure 4b[iv]).

Next, we extended the fiber‐spinning strategy to generate precise 3‐fiber nanonets (triplets) that provided the symmetric same‐diameter outer fibers and a mismatch diameter inner fiber (200–800–200 nm and 800–200–800 nm, Movies S6 and S7, Supporting Information, respectively) that resulted in symmetric cell shapes (Figure 4c[i,ii]). However, we noted that the shape of the nucleus on outer fibers was flattened and at inner fiber had nucleus distortions (invaginations). Visualizing nuclei's confocal cross sections (*yz*), immunostained for Lamin A/C (nuclear envelope marker), revealed several interesting aspects of the overall nuclear geometry. First, the nucleus thickness observed on these mismatched 3‐fiber nanonets demonstrated a similar trend compared to the same diameter 2‐fiber nanonets. Nuclei in cells on 200–800–200 nm nanonets were significantly thicker than those on 800–200–800 nm, just as the nucleus thickness in 200–200 nm nanonets was higher than 800–800 nm nanonets (Figure 4c[iii]). Second, in 3‐fiber triplets, we observed the extent of invagination in the 200–800–200 nm combination was more significant due to lower force exertion of the outside 200 nm diameter fibers (Figure 4c[iii]). Overall, our data suggest that nucleus shape, YAP localization, and nucleus invaginations are regulated by the diameter combinations of fibers external to the cell.

### 2.5 Single Fibers Sculpt Curvature‐Dependent Nuclear Invaginations

**Figure 5:**
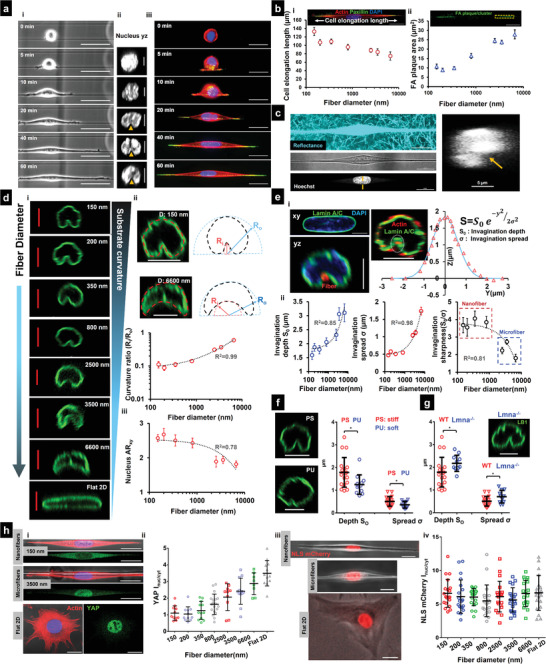
Sculpting nucleus invaginations using the curvature of single fibers. a[i]) Time‐lapse images showing different phases of cell spreading on a 1‐fiber system causing cells to spread in elongated spindle shapes (Scale bars = 50 µm). [ii] Confocal side views of representative cells at various spreading time points demonstrate the fiber–mediated nuclear deformations (yellow arrowheads, Scale bars = 5 µm). [iii] Cytoskeletal and focal adhesion arrangement in spindle‐shaped cells at various timepoints, actin (red), paxillin (green), and nucleus (blue) (Scale bars = 20 µm). b[i,ii]) Fiber‐diameter dependence of cell elongation length and FA plaque/cluster area (Scale bars = 20 µm). c) Representative C2C12 cell attached to single nanofibers, embedded within a 3D collagen gel. Collagen gel fibrils are visualized via confocal reflectance microscopy, and cross‐sectional views demonstrate the presence of invaginations (yellow arrow) at the local site where the nanofiber is present (Scale bars = 20 and 5 µm). d[i]) Representative images showing the effect of substrate curvature (fiber diameter) on the invagination shape/size (Scale bars = 5 µm). d[ii]) Representative images of the deformed nucleus shape on two diameters (150 and 6000 nm, Scale bars = 5 µm) and methodology for comparing invagination shapes: Fiber diameter dependence of Curvature ratio—Radius of curvature at the invagination side (*R*_i_) divided by radius of curvature at the nucleus apical side (*R*_o_), *n* = 12, 10, 17, 10, 10, 9, and 9, respectively. d[iii]) Nucleus aspect ratio (top view, *xy*) as a function of fiber diameter, *n* = 14, 10, 10, 11, 10, 6, and 6, respectively, Lamin A/C is stained in green. e[i]) Nucleus in spindle cells exhibit invaginations near sites of external fibers (red circle). Representative image showing cytoskeletal caging of the nucleus in spindle cell, actin (red), and Lamin A/C (green) (Scale bars = 5 µm). Invagination shape (shown in white circle) can be approximated with a bell curve. e[ii]) Invagination depth and spread increase with fiber diameter, while invagination sharpness decreases with increase in fiber diameter, *n* = 9–19 cells per diameter category. f) Influence of fiber stiffness in the regulation of invagination size, *n* = 19, 12 for fibers of similar diameter (≈350 nm) of PS (stiff *E* ≈ 1–3 GPa) and PU (soft *E* ≈ 1–10 MPa), respectively (Scale bars = 5 µm). g) Influence of impaired nuclear lamina on invagination size in cells attached to 350 nm diameter fibers, *n* = 19, 11 for control, Lamin A/C KD, respectively (Scale bars = 5 µm). h[i]) Representative stained images showing YAP localization in cells on nanofibers, microfibers, and Flat 2D (Scale bars = 20 µm). h[ii]) Comparison of YAP localization (*I*_nuc_/*I*_cyt_) in cells on different fiber diameters and Flat 2D, *n* = 11, 11, 12, 19, 10, 12, 8, and 12 cells, respectively. h[iii]) Representative images of cells expressing NLS mCherry, showing primary nuclear localization of NLS on nanofibers, microfibers and flat 2D (Scale bars = 20 µm). h[iv]) Comparison of NLS localization (*I*_nuc_/*I*_cyt_) in cells on different fiber diameters and Flat 2D, *n* = 20 cells for each category. All *R* ^2^ values shown are calculated for linear fits. Images and data are shown for C2C12 myoblasts.

Our observation that single fibers induced nucleus invaginations running along the nuclei length prompted us to investigate the role of curvature in the physical indentation of the nucleus. To interrogate this behavior, we utilized a simplified model system of a single cell spreading on a single fiber (**Figure** **5**a and Movie S8, Supporting Information). Immunostaining for the focal adhesions (paxillin) revealed that similar to the parallel‐cuboidal shape cells spread on 2‐fiber nanonets, major focal adhesion clusters localized to the poles of the spindle shape cells (Figure 5a,b and Figure S10a[i], Supporting Information). We confirmed that the cell spreading and FA cluster formation was similar to our prior observations on 2‐fiber nanonets (Figure S10b[i,ii], Supporting Information). Next, we varied the fiber diameter over a broad range (≈150 nm (high curvature) to >6000 nm (low curvature) to recapitulate the sizes of individual fibrils and larger fiber bundles observed in vivo. With the increase in diameters, we observed a drop in the cell elongation length (Figure 5b[i]) and cell aspect ratios (Figure S10c, Supporting Information), with cells adhering to the 150 nm diameter fibers forming long protrusions and having the highest aspect ratios. Analysis of the sizes of major focal adhesion clusters, demonstrated an increase at higher fiber diameters (Figure 5b[ii]). We also observed that majority of the actin stress fibers emanating from these focal adhesion clusters, caged the nucleus (Figure S10a[ii], Supporting Information), causing local invaginations in the nucleus (Figure 5d[i]). We also utilized nanofiber networks embedded in 3D collagen gels and observed that cells can maintain their elongated spindle shapes in such 3D adhesive environments and can also cause similar invaginations within the nucleus (Figure 5c and Figure S11, Supporting Information).

Varying the fiber diameter over a broad range (≈150–>6000 nm) lead to alterations in the geometric shape of the fiber‐induced local invaginations (Figure 5d[i]) in nuclei. We compared the radius of curvature (*R*_i_, Figure 5d[ii]) of the nuclear membrane at the invagination site with the radius of the best‐fit projected circle on the apical side of the nucleus (*R*_o_). With increasing diameters, we found the curvature ratio *R*_i_/*R*_o_ linearly scale with the fiber diameter. We also found the nucleus aspect ratio reduced significantly with increased fiber diameter, indicating a change in nucleus shape from elongated on small diameter fibers to flattened on larger diameter fibers (Figure 5d[iii]). Fluorescent labeling of the fiber (conjugated fibronectin, red) and the nuclear envelope (Lamin A/C, green) revealed the nucleus to be locally deformed at the location of fiber, thus allowing us to sculpt invaginations of varying sizes and shapes (Figure 5e[i]). We quantitated the effective shapes (S) of nuclear invaginations with a bell curve defined by $S=S_{0}e^{-y^{2}/2\sigma^{2}}$, where *S*_0_ denotes the invagination depth (*z*‐direction), and *σ* is related to the lateral spread (*y*‐direction) of the invagination. We found that nuclear invaginations on lower diameter fibers of high curvatures were typically ≈2 µm deep (*S*_0_) and narrow (*σ* ≈ 0.4 µm). With an increase in fiber diameters, the size (depth and lateral spread) of these invaginations was found to increase (Figure 5e[ii]). However, the sharpness ratio (ratio of *S*_0_ and *σ*) was the highest for low diameter fibers (nanofibers), which decreased with an increase in diameter, indicating smoother nuclear deformations in microfibers. Our data showed a strong linear fit between nucleus invagination shapes and fiber diameter for all tested diameters.

Next, to investigate the effect of material stiffness, independent of fiber curvature, we used polyurethane (*E*: 10–100 MPa) and polystyrene (*E*: 1–3 GPa) fibers of ≈350 nm diameters. We observed a significant decrease (Figure 5f) in the size of the nuclear invaginations in softer polyurethane fibers. We also inquired about the role of lamin A/C in nucleus invaginations and found that impairing the nuclear envelope caused by the knockdown of lamin A/C resulted in significantly larger nuclear deformations (Figure 5g). Inhibiting actin‐based contractility through Y27632 treatment resulted in thicker nuclei with significantly smaller nuclear invaginations (Figure S10b[iv], Supporting Information).

Next, we wanted to investigate the how fiber induced‐nuclear inaginations led to changes in the spatial localization of YAP. On the lower diameter nanofibers, cells primarily demonstrated a cytoplasmic YAP localization (Figure 5h[i]) with the nucleus/cytoplasmic intensity ratio less than ≈1 (Figure 5h[ii]). However, with increased fiber diameters to micron‐scale, we observed significantly enhanced YAP localization within the nucleus (Figure 5h[ii]). Interestingly, despite various levels of nuclear deformations in both nanofibers and microfibers, the nuclei demonstrated no signs of rupture, as confirmed by no appreciable leakage of nuclear localization signal (NLS) in cells expressing NLS mCherry (Figure 5h[iii] and Figure S12 and Movie S9, Supporting Information). Quantification of NLS localization revealed an average nuclear/cytoplasmic intensity ratio >5 for all fiber diameters (Figure 5h[iv]), demonstrating NLS to be primarily localized to the nucleus. We verified rupture‐free nuclear deformations by investigating possible discontinuities within the nuclear lamina at the invagination zone. Although analysis of Lamin A/C intensity revealed spatial variations along the nuclear contour, overall, there is no preferential decrease in the lamin A/C intensity to a value of zero (Figure S13e,f, Supporting Information) in the high‐curvature regions of the invagination zone. This suggests that in spite of the local sharp folding near the cell–fiber contact, the nuclear lamina is not discontinuous and thereby, supports our observations of no appreciable leakage of NLS to the cell cytoplasm.

**Figure 6:**
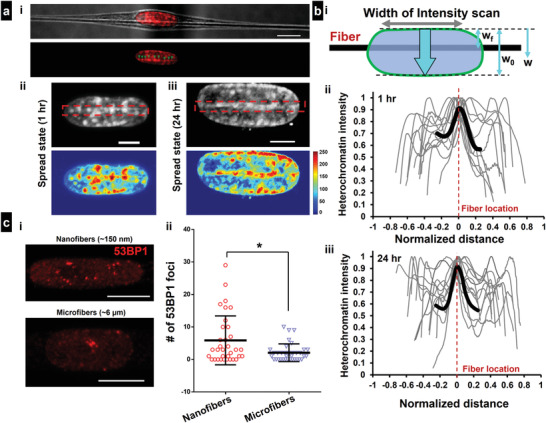
Nuclear invagination‐induced alteration in heterochromatin localization and DNA repair. a[i]) Representative spindle cell showing Heterochromatin (H3K9me3 shown in red) clusters lining preferentially along the location of the cell–fiber contact (Scale bars = 10 µm). a[ii,iii]) Representative cells in spread state: 1 and 24 h post attachment, respectively. Region of cell–fiber contact is shown with red‐dotted rectangles. Intensity heatmaps of heterochromatin localization are shown below (Scale bars = 5 µm). b[i]) Wide line scans (width of intensity scan is shown in the schematic) of heterochromatin intensity along the nuclear width, ECM fiber location is shown in black. b[ii,iii]) Intensity scans corresponding to spread cells at 1 h and 24 h post attachment, respectively, Heterochromatin intensity for each profile is normalized with respect to the maximum intensity. Normalized distance = (*w* − *w*_f_)/*w*_0_, where *w* is the distance along the nuclear width, *w*_f_ denotes the fiber location and *w*_0_ is net width of the nucleus. Average curve for the region near the cell–fiber contact is shown in black, c[i]) Representative image showing 53BP1 foci indicating sites of active DNA damage/repair on cells attached to 150 nm small diameter nanofibers (Scale bars = 10 µm). c[ii]) Quantitative analysis of 53BP1 foci on various nanofibers (sharp invaginations) and microfibers (smooth invaginations), respectively.

Nuclear deformations are usually linked with alterations in transcription regulation through chromatin remodeling and DNA damage and repair. We wanted to inquire how fiber‐induced nuclear invaginations could lead to changes in chromatin compaction and DNA repair. To this end, we performed immunofluorescent staining for methylated and acetylated chromatin markers, H3K9me3 and H3K9ac, respectively, to observe the relative localization of heterochromatin and euchromatin within the nucleus of invaginated nuclei. Interestingly, we observed increased localization of heterochromatin clusters in the vicinity of the ECM fiber, that is, the region of the invagination (**Figure** **6**a[i]). The localization was observed at both short (1 h) and long‐term (24 h post cell‐attachment) experiments (Figure 6a[ii,iii]). We quantified the preferential localization of heterochromatin clusters using two metrics: 1) 2D intensity heatmaps showing higher levels of clustering near the location of fiber, that is, the invagination regions (Figure 6a[ii]), and 2) wide intensity line scans to capture the enhanced levels of heterochromatin associated with fiber‐induced invaginations (Figure 6b). The peaks observed in the intensity scans along the nuclear width confirm the higher levels of heterochromatin clusters in the region of the nuclear invagination (Figure 6b[i,ii]). Since nanofibers and microfibers induced significantly different invagination shapes (Figure 5e[ii]), we inquired if that influenced chromatin characteristics. We observed that cells with sharper invaginations formed on lower diameter nanofibers have significantly higher number of heterochromatin clusters as compared to cells with smoother invaginations formed on larger micron scale fibers (Figure S14, Supporting Information). Concomitantly, we found a decrease in the euchromatin (H3K9ac) intensity in cells attached to nanofibers as compared to microfibers.

Next, we investigated how the shape of nuclear invaginations can impact localization of active sites of DNA damage and repair. Using the well‐established 53BP1 marker (Figure 6c[i]), we observed that the number of major sites of DNA repair increased significantly in cells attached to single nanofibers (sharper invaginations) as compared to microfibers (smoother invaginations, Figure 6c[ii]).

### 2.6 Multiple Fibers of the Same Diameter Sculpt Nucleus Shapes

**Figure 7:**
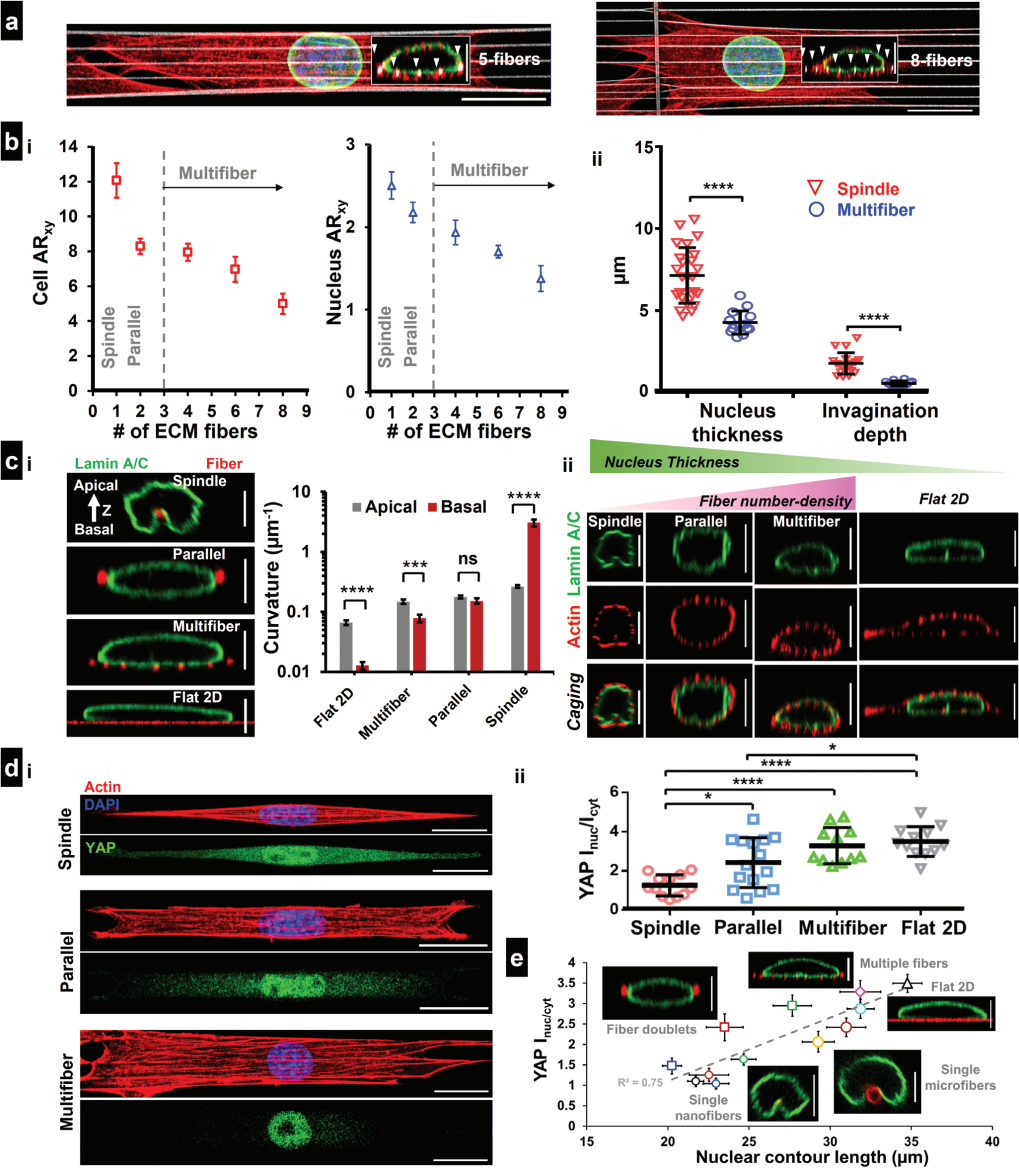
Nuclear geometry and YAP localization in cells attached to multiple fibers. All figures are for 350 nm diameter fibers. a) Representative images of cells attached to 5 and 8 fibers, respectively, stained for actin (red), Lamin A/C (green), and nucleus (blue). Fibers are coated with rhodamine fibronectin (pseudo‐colored white), Insets are cross sections (*yz*), showing the cytoskeletal caging of the nucleus with fibers identified with white arrowheads (Scale bars = 20 and 5 µm for main and inset images, respectively). b[i]) Comparison of the cell and nuclear aspect ratio (*xy* top view) as a function of the number of interacting fibers, *n* = 10, 24, 6, 7, and 5, and b[ii]) nucleus thickness (*n* = 25, 14) and invagination depth (*n* = 19, 14) in spindle (1 fiber) versus multifiber (≥3 fibers) systems. c[i]) Representative nuclear cross sections (*yz*) demonstrating shape sculpting over different substrates, Analysis of curvatures at the apical and basal side of the nucleus for the different substrates. Note, only curvature magnitude is considered here with spindle basal curvature being inward (opposite sign compared to all other categories, *n* = 11, 16, 11, and 18 for Flat 2D, multifiber, parallel [2‐fiber], and spindle [1‐fiber], respectively). c[ii]) Representative images showing the caging effect and nuclear compression as a function of fiber‐number density, Flat 2D is shown for reference, actin (red), Lamin A/C (green) (Scale bars = 5 µm). d[i,ii]) Comparison of YAP localization between spindle, 2‐fiber, multifiber systems, and flat 2D, *n* = 12, 15, 11, and 12, respectively. Representative cells are stained for actin (red), YAP (green), and nucleus (blue), (Scale bars = 20 µm). e) Combined data for YAP localization across all categories showing positive correlation between YAP localization and increase in nucleus contour length (*n* = 8–16 cells for each substrate category). Small and large circles represent single nanofibers and microfibers, respectively. Squares represent fiber doublets (same diameter and mismatch), polygons represent 3‐fiber mismatch and multifiber configurations, and Flat 2D is marked with triangle. Scale bars = 5 µm. Images and data are shown for C2C12 myoblasts.

Since single fibers were causing significant invaginations in the nuclei, we inquired if multifiber networks (≥3) of the same diameter caused multiple similar‐sized invaginations. We deposited 350 nm diameter fibers at low (≈3 µm) inter‐fiber spacing (**Figure** **7**a, images shown for single‐cell attached to 5 and 8 fibers). We found that cell and nucleus aspect ratios (at the spread state) were reduced with the increase in the number of fibers (Figure 7b[i], Movie S10, Supporting Information, for 3 fibers, and Movie S11, Supporting Information, for 7 fibers). Also, the increased compression mediated by the cytoskeletal caging caused a significant reduction in nucleus thickness and invagination depth (Figure 7b[ii]). Additionally, these deformations failed to generate ruptures in the nucleus, as confirmed by the spatial localization analysis of NLS (Figure S12, Supporting Information). We also quantified the shapes of nuclei in cells attached to flat 2D, 1‐fiber, 2‐fiber, and multiple‐fiber systems by quantifying the curvature of the nuclear envelope on either side (Figure 7c[i]). Consistent with previous literature,^[^ ^47^, ^54^ ^]^ our results indicate that the nucleus is mostly pancake‐shaped on flat continuous surfaces, which differs from cells on suspended 2‐fiber parallel doublets, where the nucleus is mostly ellipsoid shaped, with equivalent curvatures on both sides. In multifiber networks, we found the nucleus shape to have a flatter surface (low curvature) at the basal side, primarily due to cell attachment to multiple underlying fibers. In comparison, the fiber‐induced invaginations in the spindle‐shaped cells result in a much sharper curvature (inward) at the basal side. Concurrent with our previous findings that increasing the number of fibers results in increased cell contractility,^[^ ^42^ ^]^ we found that for the same diameter, increasing the number of fibers results in a decrease in nucleus thickness (Figure 7c[ii]).

Interestingly, enhanced nuclear compression caused by cell attachment to multiple fibers led to a significant increase in YAP's nuclear translocation compared to spindle and parallel‐cuboidal cells attached to 1 or 2 fibers of the same diameter, respectively (Figure 7d[i,ii]). To understand how different fiber configurations led to the altered nuclear entry of YAP, we computed the stretching of the nuclear contour (*yz* cross section) due to local invaginations (single fibers) or compression (fiber doublets), or both (multiple fibers). We observed a distinct increase in the contour length in cells attached to micron‐scale fibers or multiple fibers. In general, nuclear YAP localization for different fiber configurations and Flat 2D demonstrated a strong dependence on the nuclear contour length (*R* ^2^ = 0.75, Figure 7e).

Overall, our observations show that the organization of contractile f‐actin network in spindle cells on single fibers induces nucleus invaginations in a diameter dependent manner, while the same f‐actin network in cells attached to multiple fibers leads to higher levels of nuclear compression but reduced local invaginations and causes distinct YAP translocation to the nucleus. Irrespective of the shape, sharpness, and number of invaginations, we find that nuclei of cells attached to fibrous matrices remain rupture‐free.

## 3 Discussion

Nucleus shapes have been studied in a wide range of in vitro settings, but the role of physiological fibrous ECM such as those found in skeletal muscle tissue and around metastatic tumors,^[^ ^26^, ^27^, ^55^ ^]^ in controlling nucleus shapes remain poorly described. Fibrous ECM environments are composed of a mix of diameters (individual nanoscale fibrils that combine into larger hundreds of nanometer or micron‐scale bundles) distributed in a wide range of orientations and inter‐fiber spacing. In ECM regions with a large number of contact sites (small pore size), cells sense confinement, and with few contact points (large pore size), cells make contact with only single fibers.^[^ ^32^, ^56^, ^57^ ^]^ Cell contractility is altered with differing contact points across multiple diameters,^[^ ^38^ ^]^ resulting in changes in the overall cell^[^ ^37^, ^39^ ^]^ and nucleus shape. Here, we explore the mechanosensitivity of cells to fibrous microenvironments using nanofiber networks of precisely controlled geometries and fiber diameters. Our method of precisely controlling the external fibrous environment led to exquisite sculpting of the nucleus with and without invaginations across multiple cell lines (C2C12 mouse myoblasts, HT1080 human fibrosarcoma, and HeLa cells, Figure S15, Supporting Information). Irrespective of the shape or sharpness of the invaginations, we found nuclei to remain rupture‐free. Furthermore, fiber network organization and fiber diameter driven contractility regulates translocation of mechanosensitive transcription factors YAP/TAZ and MKL1 to the nucleus.

To understand fiber diameter‐driven contractility and associated nucleus shape changes, we estimated the increase in contractility as rounded cells spread along the fiber doublets. While the cell spreading behavior on fibers was similar to reported flat 2D behavior,^[^ ^58^ ^]^ the spreading rates were fiber diameter dependent, with cells attached to thinnest fibers achieving steady‐state spread areas the earliest. In the spread state, although actin, microtubules, and vimentin localize at the apical side in cells on 2D, we found them to cage the nucleus with no apical‐basal preference in suspended cells. Even in cells spreading on single or multiple fibers, where cell contact regions with fibers act as pseudo‐basal regions, we observed a uniform distribution of Lamin A/C expression (Figure S10c[iii], Supporting Information). Our findings on cytoskeletal caging of the cell nucleus can be further extended to 3D adhesive spaces, since caging was observed in cells adhering to nanofiber networks which are embedded in 3D collagen (Figure S2, Supporting Information).

A previous study has reported how cytoskeletal tension present on the top of the, drive the primarily apical localization of Lamin A/C in cells attached on flat surfaces.^[^ ^47^ ^]^ It is also well‐appreciated from a previous study, that the actin cytoskeletal organization is significantly influenced by the nuclear lamins, in particular Lamin A.^[^ ^59^ ^]^ To test if cytoskeletal caging is responsible for uniform distribution of Lamin A/C, we perturbed the actin cytoskeleton (through cytochalasin D, Figure 3b) and lamin A/C organization (through knockdown, Figure S9, Supporting Information). We observed that when actin cytoskeleton‐based nuclear caging is disrupted, the nuclear compression in spread cells is significantly lost. However, with Lamin A/C knockdown, the cytoskeletal caging of the nucleus is still present (Figure S9, Supporting Information). This led us to infer that cytoskeletal caging in fibrous microenvironments was the potential contributor for the uniform distribution of nuclear lamins.

To characterize the cytoskeletal tension and the cell‐ECM forces we investigated the organization and sizes of the focal adhesions in various fiber network geometries. In all diameter combinations of the fiber networks, focal adhesions were preferentially localized to the poles as cells spread on the fibers, consistent with their localization pattern during spreading at the peripheries in cells on flat 2D and the poles in cells on micropatterned substrates.^[^ ^60^ ^]^ The clustering of focal adhesions to the poles occurred early in the cell spreading phase (≈20 min), resembling those observed in 3D cell‐derived matrices.^[^ ^61^, ^62^ ^]^ Additionally, our observations that the lengths of FA clusters at the poles and the number of focal adhesions along the cell–fiber contact length increased with fiber diameter may potentially explain the slower spreading observed on larger diameter fibers.

Cell contractility increased with diameter, and we compared forces, estimated using NFM with other force measurement techniques using two metrics: traction stress and tension per stress fiber. Assuming that the forces are uniformly distributed over the entire length of FA cluster, then the average stress per FA cluster (Force/FA cluster_area_, where FA cluster_area_ = FA cluster_length_ × Diameter_fiber_) was found to be ≈8, 9, and 7 nN µm^−2^ for 200–200, 350–350, and 800–800 nm doublets, respectively, which is similar to the reported values of stress per focal adhesion in the literature (≈6 nN µm^−2^).^[^ ^63^, ^64^ ^]^ We approximated the tension in individual stress fibers by assuming their arrangement to be similar to be mechanical springs in parallel. Dividing the force per FA cluster at spread state (60 min time point) with the number of stress fibers associated with each cluster, we obtained the tension in each stress fiber to be ≈7, 11, and 15 nN for 200–200, 350–350, and 800–800 nm doublets, respectively, which agree with reported values (2–10 nN).^[^ ^65^, ^66^ ^]^ The ability to estimate spreading forces allowed us to examine the extent of nucleus compression as a function of fiber diameter. Since 800–800 nm doublets were exerting the highest forces, not surprisingly, the nuclei in these networks had the highest compression that matched the thickness of nuclei on flat 2D.^[^ ^54^, ^67^ ^]^ Loss of actin tension via cell treatment with cytochalasin D alleviated nucleus compression, a behavior consistent with previous findings.^[^ ^68^ ^]^ In contrast, the loss of microtubules via nocodazole treatment did not affect the nucleus compression. On the other hand, reduction in stiffness of the nucleus and lamin A/C KD resulted in increased compression while stiffening the nucleus reduced nucleus compression. Previous studies have shown that nucleus flattening causes stretching of the nuclear pore complexes,^[^ ^12^ ^]^ which lead to the nucleo‐cytoplasmic shuttling of various transcription factors, including YAP/TAZ^[^ ^12^ ^]^ and HDAC3.^[^ ^14^ ^]^ Across the tested diameters, our studies concluded that YAP localization increased with cell spreading due to increasing contractility. Importantly, our studies showed that for similar levels of contractility but different spread areas, YAP translocation remained similar. Additionally, our findings are not only restricted to YAP but also demonstrated a similar increase in nuclear entry for other mechanosensitive transcription factors like MKL1.

We also generated in vitro fibrous environments of a mix of diameter combinations to mimic ECM heterogeneous fibrous environments. First, we generated 200–800 nm fiber diameter mismatch doublets to study altered cell spreading, contractility, and nucleus shape. Unlike FA clustering patterns observed during cell spreading on homogenous doublets (200–200 or 800–800 nm), unexpectedly, we found a higher density of focal adhesion sites on the 200 nm diameter that matched the adhesion sites' pattern on the 800 nm diameter fiber, resulting in delayed cell spreading. Moreover, the 200 nm diameter fibers deflected more than the corresponding deflection in a 200–200 nm homogenous doublet, resulting in increased contractility. Confocal side views (*yz*) in mismatch doublets showed the nuclei to have a remarkable teardrop shape (≈60% cases), with the broad end toward the 200 nm diameter. Intensity analysis of f‐actin stress fiber distribution across the cell body in mismatch doublets revealed a higher density of stress fibers toward the 800 nm diameter fiber, thus contributing to the increased compression of the nucleus. We then generated triplets composed of two same diameter outer fibers and a third middle fiber of different diameters (200–800–200, and 800–200–800 nm). We observed the 800 nm diameter fiber to induce large invaginations in the nucleus in 200–800–200 nm triplets, consistent with the finding that the outer larger diameter pairs caused significant nucleus compression due to higher contractility. The effect of middle fiber in either of these triplets did not affect cell spreading behavior.

Nucleus invaginations have been previously reported using micropillars at sites of contact.^[^ ^69^, ^70^ ^]^ In contrast, here, we demonstrate that the invagination in the nucleus occurs across the entire length of the nucleus. Furthermore, nuclear invaginations were observed even under conditions where cells are attaching to fiber networks, embedded with 3D collagen gels (Figure S11, Supporting Information), thereby generalizing our findings to physiologically relevant 3D fibrous microenvironments. Since ECM fibers directly contributed to such deformations, naturally, we inquired if these invaginations' size depended on the fiber diameter. Our earlier work has shown that cells attached to ≈100 nm diameter fibers remain rounded, while those attached to larger diameters form elongated spindle shapes.^[^ ^34^ ^]^ Thus, we chose the fiber diameters to range from 150 to 6600 nm. Immunostaining for actin and paxillin revealed that actin stress fibers connecting the FA clustering zones straddled the nucleus, thus causing compression of the nucleus and invaginations. The high curvature (low diameter) fibers caused the sharpest invaginations (*S*_0_/*σ*), which transitioned to smooth, wider invaginations with an increase in diameter. Despite such extreme nuclear invaginations during cell spreading, no nuclear rupture events were detected (Movie S9, Supporting Information). Recently, single migrating cells have been shown to undergo nucleus rupture when they are subjected to confinement of ≈3 µm.^[^ ^71^ ^]^ On the other hand, our studies were able to get the nucleus to compress to a height of ≈4 µm. Under these conditions, we show that while fiber curvature and compression directly contribute to YAP localization, they are insufficient to cause nucleus rupture. Increasing the number of fibers of the same diameter (350 nm) further decreased the nucleus' height due to increased contractility, resulting in reduced invagination depth compared to a single 350 nm diameter fiber. Contour length, a measure of the stretching of the nucleus, shows a positive correlation with YAP localization across the different diameters and inter‐fiber spacing tested in our study (Figure 7e). In all the combinations tested by us, nuclei remained rupture‐free during cell spreading.

Previous studies have indicated how perturbation/knockdown of lamin A/C can lead to nuclear membrane instability and potential rupture.^[^ ^72^, ^73^ ^]^ The overall nuclear stability is highly dependent on the cell type, with cancer cells more vulnerable to nuclear rupture as compared to healthy cells. We performed experiments with C2C12 Lamin KD cells expressing NLS mCherry on various fiber network geometries and interestingly, we found that nuclear rupture remained rare with Lamin A/C KD in myoblast cells (Figure S13d and Movies S12 and S13, Supporting Information). It is now widely accepted that extreme physical confinement is required for nuclear rupture and the nuclear envelope is capable of unfolding and expanding under confinement‐induced compression to maintain volume, and only ruptures once volume regulation is disrupted at 3 µm confinement.^[^ ^74^ ^]^ Thus, not surprisingly when we cultured C2C12 cells within microchannels we observed instances of nuclear rupture (Figure S13c and Movie S14, Supporting Information). Our observations that nuclear lamina is not discontinuous at the invagination sites, are in good agreement with a previous study using high resolution electron microscopy and super‐resolution microscopy, that has also demonstrated that there are no lamina discontinuities even with deep nuclear invaginations induced by cytoskeletal filaments.^[^ ^75^ ^]^

Nuclear deformations induced by environmental signals, have been previously associated with rapid (within few hours) epigenetic changes, including upregulation of DNA methylation.^[^ ^14^ ^]^ Our results demonstrate that fiber‐induced nuclear inaginations mediate preferential organization of heterochromatin clusters locally near the invagination zone at short and long durations. The localization pattern can be attributed to the inward folding of the nuclear lamina similar to the formation of lamina‐associated domains.^[^ ^76^ ^]^ Apart from inducing preferential patterns of hetrochromatin, the overall shape of the invagination also appears to play a role in the extent of DNA damage and repair. Our findings that DNA repair is enhanced in cells on smaller diameter nanofibers (induce sharper invaginations on high curvature) is in agreement with literature showing high curvature regions of the nuclear envelope leads to DNA damage.^[^ ^9^ ^]^

## 4 Conclusion

In conclusion, we demonstrate sculpting of the nucleus and their functional response using in vivo mimicking fibrous environments through regulation of cell contractility in a fiber‐diameter and number‐density manner. The nuclei remain robust mechanical organelles capable of withstanding extensive shape changes and invaginations without undergoing rupture. Nuclear invaginations have enrichment of heterochromatin clusters along the length of invagination, suggestive of epigenetic alterations. We envision that control of nuclear 3D shape in these environments will yield new fundamental insights on the ECM‐mediated alterations in the sub‐nuclear chromatin organization and overall gene expression during cancer metastasis, wound healing, cell differentiation, and myogenesis.

## 5 Experimental Section

### Fabrication of Nanofiber Networks

Using the previously reported non‐electrospinning STEP technique,^[^ ^51^, ^52^, ^53^ ^]^ suspended fiber nanonets (horizontal arrays of densely spaced (≈12 µm) nanofibers of differing diameters (200, 350, 800 nm) deposited on widely spaced (≈250 µm) vertical support fibers (≈2 µm diameter), were manufactured from solutions of polystyrene (MW: 2 000 000 g mol^−1^; Category No. 829; Scientific Polymer Products, Ontario, NY, USA) was dissolved in xylene (X5‐500; Thermo Fisher Scientific, Waltham, MA, USA) in 7–13 wt% solutions. Micron scale (≥2 µm) fibers were manufactured from 2 to 5 wt% of high molecular weight polystyrene (MW: 15 000 000 g mol^−1^, Agilent Technologies, Santa Clara, CA, USA). Briefly, the polymeric solutions was extruded through a micropipette (inside diameter: 100 µm; Jensen Global, Santa Barbara, CA, USA) for deposition of aligned fibers on a hollow substrate. Measured (using scanning electron microscopy images) diameters for the 150, 200, 350, 800 nm, 2.5, 3.5, and 6.6 µm diameter categories were 153 ± 1.2, 206 ± 2.6, 361 ± 4.5, 808 ± 7.6 nm, 2.50 ± 0.02, 3.48 ± 0.09, and 6.62 ± 0.17 µm (values were shown as Mean ± SEM, *n* = 37, 59, 90, 92, 28, 21, and 25, respectively). For the 350 nm diameter category, fiber networks with varying spacing (3–25) µm were used. All fiber networks were cross‐linked at intersection points using a custom fusing chamber, to create fixed‐fixed boundary conditions. For embedding nanofiber networks in 3D fibrous environments, type I collagen gels (Advanced Biomatrix, Carlsbad, CA) were used. Briefly, acidic collagen solutions (bovine, 1 mg mL^−1^ final concentration) were neutralized with 0.25 m NaOH and transferred around nanofiber scaffolds attached to 6‐well culture plates. Collagen gels were polymerized for 1 h within an incubator kept at 37 °C and 5% CO_2_, followed by addition of cell‐culture medium.

### Cell Culturing and Experimental Procedure

C2C12 mouse myoblasts (ATCC), HT1080 human fibrosarcoma, and HeLa cells were cultured in Dulbecco's modified Eagle's medium (Invitrogen, Carlsbad, CA, USA) supplemented with 10% fetal bovine serum (Gibco, Thermo Fisher Scientific) in T25 flasks (Corning, Corning, NY, USA) kept at 37 °C and 5% CO_2_ in a humidified incubator. For imaging cell spreading dynamics scaffolds containing fiber nanonets were mounted in glass‐bottom single well plate. For immunofluorescent staining experiments, cell seeding was performed on fiber scaffolds mounted on glass‐bottom six‐well plates (Cellvis, Mountain View, CA, USA). Prior to experimentation, fibers were sterilized in 70% ethanol for 10 min and functionalized for 1 h under incubation at 37 °C using 4 µg mL^−1^ fibronectin in PBS (Invitrogen, Carlsbad, CA, USA). For fluorescent labeling of fibers, rhodamine conjugated fibronectin (Cytoskeleton Inc., Denver, CO, USA) was used with the same concentration and incubation time. Before cell seeding, the test platform was moved into an AxioObserver microscope under incubation conditions of 37 °C and 5% CO_2_ (Zeiss, Oberkochen, Germany). Without disturbing the closed environment, a droplet (≈100 µL) of cell suspension was deposited on the fiber nanonets to begin the experiment.

### Live Imaging

Time‐lapse optical imaging was started immediately before introducing the cell suspension to the fibers. Imaging was performed at 20 × 0.8 NA objective in a Zeiss AxioObxerver Z1 microscope with an interval of 1–3 min for 1 h. C2C12 or HT1080 cells expressing NLS mCherry were imaged every 3 min with a TRITC filter set. For observing nuclear rupture events in the presence of physical confinement, cells moving within microchannels were considered. Briefly, PDMS devices with an array of 10 × 3 µm (*W* × *H*) microchannels were fabricated as previously described.^[^ ^15^, ^77^, ^78^ ^]^ C2C12 NLS‐mCherry cells were seeded in the devices in serum‐free DMEM. The top four wells were filled with DMEM + 10% FBS to create serum gradient for cells migrating through channels. Live images were captured on a Nikon A1 confocal microscope every 5 min.

### Immunofluorescent Staining and Imaging

Cells were fixed with 4% paraformaldehyde for 15 min at various timepoints (5, 10, 20, 40, and 60 min), following initial cell fiber contact. Cells were then permeabilized with a 0.1% Triton X‐100 solution, washed in PBS twice and blocked with 5% goat serum (Invitrogen, Grand Island, NY) for 30 min. Primary antibodies, diluted in an antibody dilution buffer consisting of PBS with 1% bovine serum albumin and Triton‐X 100, were added to the fixed cells and kept either 1) overnight at 4 °C or 2) at room temperature for 3 h or 3) at 37 °C for 2 h. Diluted secondary antibodies, along with the conjugated Phalloidin‐TRITC (Santa Cruz Biotechnology, Dallas, TX, USA) or Alexa Fluor 647 Phalloidin (Invitrogen) diluted in 1:80 ratio, were subsequently added and stored in a dark place for 45 min. Following a three times PBS wash, DAPI (4′,6‐diamidimo‐2‐phenylindole) or Hoechst 33342 (Thermo Fisher Scientific) was added for 5 min to stain the cell nuclei. Primary antibodies include Anti‐Vimentin antibody (1:250, rabbit monoclonal, EPR3776, Abcam), Anti‐phospho‐Paxillin (1:100, rabbit polyclonal, pTyr31, Invitrogen), Anti‐beta tubulin (1:500, mouse monoclonal, 2 28 33, Invitrogen), Anti‐Lamin A/C (1:1000, mouse monoclonal, sc‐376248, Santa Cruz Biotechnology), Anti‐Lamin B1 (1:500, mouse monoclonal, sc‐374015, Santa Cruz Biotechnology), Anti‐YAP (1:100, mouse monoclonal, sc‐101199, Santa Cruz Biotechnology), Anti‐H3K9ac (1:100, rabbit monoclonal, #9649, Cell Signaling Technology Danvers, MA, USA), Anti‐H3K9me2/3 (1:100, mouse monoclonal, #5327, Cell Signaling Technology), and Anti‐53BP1 (1:100, rabbit polyclonal, #4937, Cell Signaling Technology). Secondary antibodies include Goat anti‐Rabbit IgG Alexa Fluor 488 (1:200, Invitrogen), Goat anti‐mouse IgG Alexa Fluor 488 (1:200–1:1000, Invitrogen), and Goat anti‐mouse IgG Alexa Fluor 647 secondary antibody (1:500, Invitrogen). Images were taken using an inverted Zeiss microscope using a 63x objective (NA 1.20, water immersion). Confocal microscopy was performed using a laser scanning confocal microscope (LSM 880, Carl Zeiss Inc.) and images were obtained using a 63 × 1.15 NA water immersion objective. Z‐stacks were taken with slice thicknesses ranging between 0.3 and 0.5 µm. Z‐stack images were processed in the Zen Blue software (Carl Zeiss Inc.). Reflectance imaging on collagen gels were performed in a Nikon Ti2 laser scanning confocal microscope and images were obtained with a 40 × 0.75 NA objective.

### Generation of Knockdown Cell Lines

Lmna‐KD were created using shRNA encoded on pLKO.1 puro plasmid (Addgene #8453) and introduced into C2C12 with second generation lentivirus. Sequences for shRNA were obtained from Broad Institute Genetic Perturbation Platform. The sequence used for this study was GCGGCTTGTGGAGATCGATAA. Viral particles were produced in HEK293T following calcium phosphate transfection using 4.8, 30, and 33.6 µg of VSVG (Addgene #14888), psPAX2 (Addgene #12260), and pLKO.1 puro Lmna‐shRNA plasmids, respectively. After 48 h, supernatant containing viral particles were concentrated 100× following 2‐h ultracentrifugation 50 000 × *g* and 4 °C. Cells were transduced in full growth media (DMEM supplemented with 10% FBS) containing viral particles for 48 h and verified (Figure S16, Supporting Information) via western blot using anti‐Lmna/c mouse mAb (Cell Signaling Technology #4777).

### Pharmacological Inhibition

For cytoskeletal inhibition, cells in suspension were incubated with 2 µm cytochalasin D (actin disruption) (Fischer Scientific), 1 µm nocodazole (microtubule disruption) (Sigma Aldrich), and 10–20 µM Y27632 (ROCK inhibition) (Hellobio, Princeton, NJ, USA) for 30 min–1 h. For chromatin compaction/decompaction, cells were pre‐treated with histone demethylase inhibitor (Methylstat, 5 µm, 48 h incubation) (Sigma Aldrich, St. Louis, MO, USA) and HDACi VPA (2 mm, 24 h incubation) (Sigma Aldrich), respectively. At least *N* = 2 replicates were performed for each drug condition.

### Analysis of Shape Metrics

Cells in suspension demonstrated a rounded morphology which evolves as they attach and spread on a substrate. Cell circularity was defined by the relation:

(1) $$\mathrm{Circularity}=4\pi A/P^{2}$$

where *A* was spread area (µm^2^) and *P* was perimeter (µm). A perfect circle resulted in a circularity value of 1.0. As the shape elongates, this value approached 0. Polarized cells were manually outlined in ImageJ and the aspect ratio was quantified using the Bounding Rectangle Function.

### Analysis of Focal Adhesion Clusters and Actin Stress Fibers

Consistent with the authors' previously reported convention, the length of the focal adhesion (FA) cluster was quantified as the longest continuous length of immunostained paxillin. Intensity profiles for paxillin were generated by performing line scans along the cell–fiber contact region. For averaging purposes, profiles were normalized in two steps: 1) Intensity (plotted along *y*‐axis) was normalized with respect to peak intensity of the corresponding profile and 2) cell length (plotted along *x*‐axis) was normalized with respect to the net length (measured from end‐to‐end) of the particular cell. Intensity profiles were subsequently averaged using a custom MATLAB routine, using a bin width of 0.01. Maximum intensity projections of phalloidin stained cells were utilized to count the number of major actin stress fibers emanating from each FA cluster and in the perinuclear region. Orientation of the actin stress fibers were measured with respect to the undeflected nanofiber orientation. For average actin localization in Figure 4b, images of perinuclear regions (17 cells) were averaged using a custom MATLAB routine, and the average image is represented as an intensity heatmap.

### Analysis of Nucleus Shapes and Deformations

Confocal z‐stacks of DAPI/Hoechst/Lamin stained nuclei were processed in either ImageJ (NIH; https://imagej.nih.gov/ij/) or Zen Blue (Carl Zeiss Inc). Nucleus projected area was calculated from the top (*xy*) view at the equatorial plane (plane of the nanonets). The shape (*xy*) of the nucleus was approximated as an ellipse and quantified by the ellipse eccentricity.

(2) $$\mathrm{Eccentricity}={\left(1-b^{2}/a^{2}\right)}^{0.5}$$

where 2a and 2b represented the shape of the major and minor axis of the ellipse, respectively. The eccentricity value of a perfectly symmetrical circle was 0, while the value increased to 1, with increasing elongation of the ellipse. Nucleus aspect ratio was measured by manually outlining and using the Bounding Rectangle Function in ImageJ.

Nucleus thickness was measured from the orthogonal side views (*xz* or *yz*) generated with the *Ortho* function in Zen Blue, and the maximum *z*‐dimension was considered as the thickness of the nucleus. To quantify nuclear shapes in “mismatch” diameter fiber doublets, cross‐sectional side views (*yz*) were generated using *Ortho* function in Zen Blue. A line was then drawn through the nucleus mid‐width as shown in (Figure 4b[iii]) and the area of the half cross sections were measured and designated as *A*_max_ and *A*_min_ based on larger and smaller area, respectively. NAI was defined by *A*_min_/*A*_max_. Thus symmetric nuclei had a NAI of ≈1, while teardrop shaped nuclei has a NAI <1.

To quantify nuclear invaginations in spindle shaped cells attached to single fibers, the deformed region of the nuclear envelope (*yz* cross‐sectional view) was manually outlined in ImageJ and a bell curve ($S=S_{0}e^{-y^{2}/2\sigma^{2}}$, Figure 5e) was fitted to the manual trace using a custom MATLAB (https://www.mathworks.com/) code to evaluate the individual parameters *S*_0_ (invagination depth) and *σ* (invagination spread). To quantify the level of curvature of the nuclear surface, a best‐fit circle was placed at the apical and basal surface of the cross‐sectional view (*yz*) of the nucleus, in the Zen Blue software. The curvature was defined as the inverse of the radius of this best‐fit circle.

### Fluorescent Intensity Analysis

To compare the basal and apical localization of cytoskeletal and nuclear envelope components on fiber doublets, the orthogonal side views of the respective channels were first generated in Zen Blue. Rectangular intensity profiles with a 5 µm width were computed at the cell/nucleus mid‐width (Figure 1e). The peak intensities at the apical and basal side were subsequently extracted to compute the intensity ratio between basal and apical side.

To quantify the YAP/NLS localization within cells, cells and nuclei were first manually outlined in Zen Blue to get the mean YAP/NLS intensity within the whole cell (*I*_cell_) and inside the nucleus (*I*_nuc_) and the corresponding projected areas (*A*_cell_ and *A*_nuc_). The average intensity within the cytoplasm (projected area: *A*_cyt_) was indirectly quantified from the following relations:

(3) $$I_{\mathrm{cell}}\times A_{\mathrm{cell}}=I_{\mathrm{nuc}}\times A_{\mathrm{nuc}}+I_{\mathrm{cyt}}\times A_{\mathrm{cyt}}$$

(4) $$A_{\mathrm{cell}}=A_{\mathrm{nuc}}+A_{\mathrm{cyt}}$$

The intensity ratio *I*_nuc/cyt_ was quantified by the ratio of the average intensities within the nucleus (*I*_nuc_) and the cytoplasm (*I*_cyt_).

For NLS intensity analysis (Figure S12, Supporting Information), rectangular intensity profiles (1 µm width) were taken along the cell length. Fluorescent intensity was normalized with respect to peak intensity of the corresponding profile and centered with respect to the nucleus midpoint.

### Calculation of Cell Adhesion Forces

Cell–fiber adhesion forces were quantified using a previously reported NFM technique.^[^ ^38^, ^41^, ^79^ ^]^ To implement NFM nanofiber networks consisting of large diameter (2 µm) support fibers placed ≈250 µm apart and an orthogonal layer of small diameter (200–800 nm) fiber layer, with both fiber layers fused at the junctions using solvent vapor. This generates fixed–fixed boundary conditions at both ends of the small diameter fibers. Additionally since focal adhesion organization demonstrates distinct clustering at the cell poles, the force exertion on each fiber could be considered as a 2‐point load. Taut nanofibers were thus approximated as loaded Euler Bernoulli beams with fixed–fixed boundary conditions and with forces exerted at either cell pole at an angle. This direction of force exertion was taken along the average orientation (with respect to the undeflected fibers) of the f‐actin stress fibers emanating from the polar FA clusters. To quantify exerted forces, cell force‐mediated fiber deflections were analyzed and these deflection profiles of fibers bonded to the support fibers on either end, serve as input for the optimization framework in MATLAB utilizing the gradient based optimizer.

The formulation for the cell forces were reported previously by the authors.^[^ ^40^ ^]^ Briefly each taut fiber could be estimated as a beam with an axial force. Finite element method could be used discretize the beam into a finite number of uniform straight beam elements as shown in this schematic. Shear forces and moments at two nodes were shown by *Y*_1_, *M*_1_, *Y*_2_, and *M*_2_.


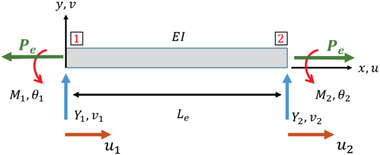
Finite beam element with axial force (*P*_e_), shear force (*Y*) and Bending moment (*M*).

Using Castigliano's theorem to model the strain energy of each beam element, one could derive the interrelationship between the shear forces and moments exerted on the beam elements and the corresponding displacements.

(5) $$\left\{F\right\}=\left[\left[k\right]+\left[n\right]\right]\left\{q\right\}$$

Here [*k*] and [*n*] were referred to as the basic stiffness matrix and incremental stiffness matrix or geometric stiffness matrix, respectively.

Here

(6) $$\begin{array}{ccc} \left\{F\right\} & = & \left\{\begin{array}{c} Y_{1}\\ M_{1}\\ Y_{2}\\ M_{2} \end{array}\right\},\left[k\right]=\frac{EI}{L_{e}}\left[\begin{array}{cccc} \frac{12}{L_{e}^{2}} & \frac{6}{L_{e}} & -\frac{12}{L_{e}^{2}} & \frac{6}{L_{e}}\\ \frac{6}{L_{e}} & 4 & -\frac{6}{L_{e}} & 2\\ -\frac{12}{L_{e}^{2}} & -\frac{6}{L_{e}} & \frac{12}{L_{e}^{2}} & -\frac{6}{L_{e}}\\ \frac{6}{L_{e}} & 2 & -\frac{6}{L_{e}} & 4 \end{array}\right],\\ \left[n\right] & = & \frac{P_{e}}{10}\left[\begin{array}{cccc} \frac{12}{L_{e}} & 1 & -\frac{12}{L_{e}} & 1\\ 1 & \frac{4L_{e}}{3} & -1 & -\frac{L_{e}}{3}\\ -\frac{12}{L_{e}} & -1 & \frac{12}{L_{e}} & -1\\ 1 & -\frac{L_{e}}{3} & -1 & \frac{4L_{e}}{3} \end{array}\right],\left\{q\right\}=\left\{\begin{array}{c} v_{1}\\ \theta_{1}\\ v_{2}\\ \theta_{2} \end{array}\right\} \end{array}$$

*P*_e_ was the axial tensile force in the element and *L*_e_ was the length of the element.

The optimization framework was based on minimizing the following objective function

(7) $$g\left(x\right)=\frac{1}{2}{\left\|V_{\mathrm{EXP}}-V_{\mathrm{FEM}}\right\|}^{2}$$

Here *V*_EXP_ represented the vector of vertical displacements generated from interpolation of the experimental vertical displacements and *V*_FEM_ was the vector of computational vertical displacements from the finite element model.

This error between them (g(x)) was minimized iteratively to generate the adhesion forces. In fiber doublets (homogenous diameters), due to the high level of symmetry in fiber deflections on either fiber, magnitude of the net cell force exerted was approximated as twice the magnitude of the cell‐adhesion force with each fiber.

### Statistical Analysis and Data Fitting

Statistical analysis was performed in GraphPad Prism (GraphPad Software, La Jolla, CA, USA) software. For statistical comparison between multiple groups, ANOVA (along with Tukey's honestly significant difference test) was utilized. For pairwise comparisons with a control group, Student's *t*‐test was used. In all scatter data column plots, error bars represented standard deviation. Unless otherwise mentioned, in all other plots, error bars represented standard error of measurement. For all statistical plots *, **, ***, and **** represented *p* < 0.05, 0.01, 0.001, and 0.0001, respectively. Data fitting was performed in either MATLAB (for nonlinear fits) or Microsoft Excel (linear fits).

## Conflict of Interest

The authors declare no conflict of interest.

## Authors Contribution

A.G. and A.T. are equally contributing second authors. A.S.N. conceived and oversaw the project. A.S.N. and K.K. designed experiments. A.J., A.T., and A.G. performed experiments. A.J., A.T., A.G., K.K., and A.S.N. analyzed data. A.J. and A.T. wrote the manuscript. R.K.K. and A.S.N. developed the cell force model. A.T., A.K., and K.K. developed the lamin deficient, and DN‐KASH2 cell lines. A.J. and A.G. developed the suspended fiber networks. A.J. and A.T. performed confocal imaging. All authors contributed to reading and editing the manuscript.

## Supporting information

Supporting Information

Supplemental Movie 1

Supplemental Movie 2

Supplemental Movie 3

Supplemental Movie 4

Supplemental Movie 5

Supplemental Movie 6

Supplemental Movie 7

Supplemental Movie 8

Supplemental Movie 9

Supplemental Movie 10

Supplemental Movie 11

Supplemental Movie 12

Supplemental Movie 13

Supplemental Movie 14

## Data Availability

The data that support the findings of this study are available from the corresponding author upon reasonable request.

## References

[1] F. Guilak , J. R. Tedrow , R. Burgkart , Biochem. Biophys. Res. Commun. 2000, 269, 781. 10720492 10.1006/bbrc.2000.2360

[2] J. Swift , D. E. Discher , J. Cell Sci. 2014, 127, 3005. 24963133 10.1242/jcs.149203 PMC4095853

[3] B. M. Skinner , E. E. P. Johnson , Chromosoma 2017, 126, 195. 27631793 10.1007/s00412-016-0614-5 PMC5371643

[4] C. Uhler , G. V. Shivashankar , Trends Cancer 2018, 4, 320. 29606315 10.1016/j.trecan.2018.02.009

[5] S. G. Alam , D. Lovett , D. I. Kim , K. J. Roux , R. B. Dickinson , T. P. Lele , J. Cell Sci. 2015, 128, 1901. 25908852 10.1242/jcs.161703 PMC4457156

[6] M. L. Lombardi , D. E. Jaalouk , C. M. Shanahan , B. Burke , K. J. Roux , J. Lammerding , J. Biol. Chem. 2011, 286, 26743. 21652697 10.1074/jbc.M111.233700 PMC3143636

[7] T. P. Lele , R. B. Dickinson , G. G. Gundersen , J. Cell Biol. 2018, 217, 3330. 30194270 10.1083/jcb.201804052 PMC6168261

[8] A. D. Stephens , P. Z. Liu , E. J. Banigan , L. M. Almassalha , V. Backman , S. A. Adam , R. D. Goldman , J. F. Marko , Mol. Biol. Cell 2018, 29, 220. 29142071 10.1091/mbc.E17-06-0410 PMC5909933

[9] Y. Xia , I. L. Ivanovska , K. Zhu , L. Smith , J. Irianto , C. R. Pfeifer , C. M. Alvey , J. Ji , D. Liu , S. Cho , R. R. Bennett , A. J. Liu , R. A. Greenberg , D. E. Discher , J. Cell Biol. 2018, 217, 3796. 30171044 10.1083/jcb.201711161 PMC6219729

[10] W. H. De vos , F. Houben , M. Kamps , A. Malhas , F. Verheyen , J. Cox , E. M. M. Manders , V. L. R. M. Verstraeten , M. A. M. Van steensel , C. L. M. Marcelis , A. Van den wijngaard , D. J. Vaux , F. C. S. Ramaekers , J. L. V. Broers , Hum. Mol. Genet. 2011, 20, 4175. 21831885 10.1093/hmg/ddr344

[11] F. Alisafaei , D. S. Jokhun , G. V. Shivashankar , V. B. Shenoy , Proc. Natl. Acad. Sci. USA 2019, 116, 13200. 31209017 10.1073/pnas.1902035116 PMC6613080

[12] A. Elosegui‐Artola , I. Andreu , A. E. M. Beedle , A. Lezamiz , M. Uroz , A. J. Kosmalska , R. Oria , J. Z. Kechagia , P. Rico‐Lastres , A. L. Le Roux , C. M. Shanahan , X. Trepat , D. Navajas , S. Garcia‐Manyes , P. Roca‐Cusachs , Cell 2017, 171, 1397. 29107331 10.1016/j.cell.2017.10.008

[13] T. J. Kirby , J. Lammerding , Nat. Cell Biol. 2018, 20, 373. 29467443 10.1038/s41556-018-0038-y PMC6440800

[14] K. Damodaran , S. Venkatachalapathy , F. Alisafaei , A. V. Radhakrishnan , D. S. Jokhun , V. B. Shenoy , G. V. Shivashankar , Mol. Biol. Cell 2018, 29, 3039. 30256731 10.1091/mbc.E18-04-0256 PMC6333178

[15] P. Mistriotis , E. O. Wisniewski , K. Bera , J. Keys , Y. Li , S. Tuntithavornwat , R. A. Law , N. A. Perez‐Gonzalez , E. Erdogmus , Y. Zhang , R. Zhao , S. X. Sun , P. Kalab , J. Lammerding , K. Konstantopoulos , J. Cell Biol. 2019, 218, 4093. 31690619 10.1083/jcb.201902057 PMC6891075

[16] B. R. Freedman , A. B. Rodriguez , R. J. Leiphart , J. B. Newton , E. Ban , J. J. Sarver , R. L. Mauck , V. B. Shenoy , L. J. Soslowsky , Sci. Rep. 2018, 8, 10854. 30022076 10.1038/s41598-018-29060-y PMC6052000

[17] P. P. Provenzano , R. Vanderby , Matrix Biol. 2006, 25, 71. 16271455 10.1016/j.matbio.2005.09.005

[18] T. A. H. Järvinen , L. Józsa , P. Kannus , T. L. N. Järvinen , M. Järvinen , J. Muscle Res. Cell Motil. 2002, 23, 245. 12500904 10.1023/a:1020904518336

[19] A. R. Gillies , R. L. Lieber , Muscle Nerve 2011, 44, 318. 21949456 10.1002/mus.22094 PMC3177172

[20] M. W. Conklin , J. C. Eickhoff , K. M. Riching , C. A. Pehlke , K. W. Eliceiri , P. P. Provenzano , A. Friedl , P. J. Keely , Am. J. Pathol. 2011, 178, 1221. 21356373 10.1016/j.ajpath.2010.11.076 PMC3070581

[21] P. P. Provenzano , D. R. Inman , K. W. Eliceiri , J. G. Knittel , L. Yan , C. T. Rueden , J. G. White , P. J. Keely , BMC Med. 2008, 6, 11. 18442412 10.1186/1741-7015-6-11 PMC2386807

[22] K. R. Levental , H. Yu , L. Kass , J. N. Lakins , M. Egeblad , J. T. Erler , S. F. T. Fong , K. Csiszar , A. Giaccia , W. Weninger , M. Yamauchi , D. L. Gasser , V. M. Weaver , Cell 2009, 139, 891. 19931152 10.1016/j.cell.2009.10.027 PMC2788004

[23] J. M. Szulczewski , D. R. Inman , M. Proestaki , J. Notbohm , B. M. Burkel , S. M. Ponik , Acta Biomater. 2021, 129, 96. 33965625 10.1016/j.actbio.2021.04.053 PMC8848478

[24] J. Starke , K. Maaser , B. Wehrle‐Haller , P. Friedl , Exp. Cell Res. 2013, 319, 2424. 23830878 10.1016/j.yexcr.2013.04.003

[25] A. D. Doyle , N. Carvajal , A. Jin , K. Matsumoto , K. M. Yamada , Nat. Commun. 2015, 6, 8720. 26548801 10.1038/ncomms9720 PMC4643399

[26] T. Ushiki , Arch. Histol. Cytol. 2002, 65, 109. 12164335 10.1679/aohc.65.109

[27] M. Fernández , J. Keyriläinen , R. Serimaa , M. Torkkeli , M. L. Karjalainen‐Lindsberg , M. Tenhunen , W. Thomlinson , V. Urban , P. Suortti , Phys. Med. Biol. 2002, 47, 577. 11900192 10.1088/0031-9155/47/4/303

[28] A. Padhi , A. S. Nain , Ann. Biomed. Eng. 2020, 48, 1071. 31485876 10.1007/s10439-019-02337-7

[29] P. Keely , A. Nain , F1000Research 2015, 4, 1408. 10.12688/f1000research.6623.1 PMC4754037 26918156

[30] K. Wolf , M. te Lindert , M. Krause , S. Alexander , J. te Riet , A. L. Willis , R. M. Hoffman , C. G. Figdor , S. J. Weiss , P. Friedl , J. Cell Biol. 2013, 201, 1069. 23798731 10.1083/jcb.201210152 PMC3691458

[31] P. Friedl , E. Sahai , S. Weiss , K. M. Yamada , Nat. Rev. Mol. Cell Biol. 2012, 13, 743. 23072889 10.1038/nrm3459

[32] A. D. Doyle , F. W. Wang , K. Matsumoto , K. M. Yamada , J. Cell Biol. 2009, 184, 481. 19221195 10.1083/jcb.200810041 PMC2654121

[33] S. Meehan , A. S. Nain , Biophys. J. 2014, 107, 2604. 25468339 10.1016/j.bpj.2014.09.045 PMC4255195

[34] B. Koons , P. Sharma , Z. Ye , A. Mukherjee , M. H. Lee , D. Wirtz , B. Behkam , A. S. Nain , ACS Nano 2017, 11, 12037. 29144730 10.1021/acsnano.7b04567

[35] A. Mukherjee , B. Behkam , A. S. Nain , iScience 2019, 19, 905. 31513975 10.1016/j.isci.2019.08.023 PMC6742781

[36] J. Singh , A. Pagulayan , B. A. Camley , A. S. Nain , Proc. Natl. Acad. Sci. U. S. A. 2021, 118, e2011815118. 33737392 10.1073/pnas.2011815118 PMC8000107

[37] A. Jana , I. Nookaew , J. Singh , B. Behkam , A. T. Franco , A. S. Nain , FASEB J. 2019, 33, 10618. 31225977 10.1096/fj.201900131R PMC6766658

[38] K. Sheets , J. Wang , W. Zhao , R. Kapania , A. S. Nain , Biophys. J. 2016, 111, 197. 27410747 10.1016/j.bpj.2016.05.031 PMC4945583

[39] K. Sheets , S. Wunsch , C. Ng , A. S. Nain , Acta Biomater. 2013, 9, 7169. 23567946 10.1016/j.actbio.2013.03.042

[40] B. Tu‐Sekine , A. Padhi , S. Jin , S. Kalyan , K. Singh , M. Apperson , R. Kapania , S. C. Hur , A. Nain , S. F. Kim , FASEB J. 2019, 33, 14137. 31657647 10.1096/fj.201900717RR PMC6894044

[41] P. M. Graybill , A. Jana , R. K. Kapania , A. S. Nain , R. V. Davalos , ACS Nano 2020, 15, 2554. 33236888 10.1021/acsnano.0c07020 PMC10949415

[42] A. Padhi , K. Singh , J. Franco‐Barraza , D. J. Marston , E. Cukierman , K. M. Hahn , R. K. Kapania , A. S. Nain , Commun. Biol. 2020, 3, 1. 32694539 10.1038/s42003-020-01117-7 PMC7374753

[43] H. Wolfenson , T. Iskratsch , M. P. Sheetz , Biophys. J. 2014, 107, 2508. 25468330 10.1016/j.bpj.2014.10.041 PMC4255173

[44] Y. Li , D. Lovett , Q. Zhang , S. Neelam , R. A. Kuchibhotla , R. Zhu , G. G. Gundersen , T. P. Lele , R. B. Dickinson , Biophys. J. 2015, 109, 670. 26287620 10.1016/j.bpj.2015.07.006 PMC4547341

[45] A. Katiyar , V. J. Tocco , Y. Li , V. Aggarwal , A. C. Tamashunas , R. B. Dickinson , T. P. Lele , Soft Matter 2019, 15, 9310. 31674621 10.1039/c9sm01666j PMC6870954

[46] S. B. Khatau , C. M. Hale , P. J. Stewart‐Hutchinson , M. S. Patel , C. L. Stewart , P. C. Searson , D. Hodzic , D. Wirtz , Proc. Natl. Acad. Sci. USA 2009, 106, 19017. 19850871 10.1073/pnas.0908686106 PMC2776434

[47] T. O. Ihalainen , L. Aires , F. A. Herzog , R. Schwartlander , J. Moeller , V. Vogel , Nat. Mater. 2015, 14, 1252. 26301768 10.1038/nmat4389 PMC4655446

[48] S. Dupont , L. Morsut , M. Aragona , E. Enzo , S. Giulitti , M. Cordenonsi , F. Zanconato , J. Le Digabel , M. Forcato , S. Bicciato , N. Elvassore , S. Piccolo , Nature 2011, 474, 179. 21654799 10.1038/nature10137

[49] J. K. Kim , A. Louhghalam , G. Lee , B. W. Schafer , D. Wirtz , D. H. Kim , Nat. Commun. 2017, 8, 1. 29242553 10.1038/s41467-017-02217-5 PMC5730574

[50] A. D. Stephens , E. J. Banigan , J. F. Marko , Curr. Opin. Cell Biol. 2019, 58, 76. 30889417 10.1016/j.ceb.2019.02.006 PMC6692209

[51] A. S. Nain , M. Sitti , A. Jacobson , T. Kowalewski , C. Amon , Macromol. Rapid Commun. 2009, 30, 1406. 21638398 10.1002/marc.200900204

[52] A. S. Nain , J. Wang , Polym. J. 2013, 45, 695.

[53] J. Wang , A. S. Nain , Langmuir 2014, 30, 13641. 25310055 10.1021/la503011u

[54] A. Buxboim , J. Irianto , J. Swift , A. Athirasala , J. W. Shin , F. Rehfeldt , D. E. Discher , Mol. Biol. Cell 2017, 28, 3333. 28931598 10.1091/mbc.E17-06-0393 PMC5687034

[55] P. G. Gritsenko , O. Ilina , P. Friedl , J. Pathol. 2012, 226, 185. 22006671 10.1002/path.3031

[56] P. Friedl , K. Wolf , J. Cell Biol. 2010, 188, 11. 19951899 10.1083/jcb.200909003 PMC2812848

[57] S. P. Carey , C. M. Kraning‐Rush , R. M. Williams , C. a Reinhart‐King , Biomaterials 2012, 33, 4157. 22405848 10.1016/j.biomaterials.2012.02.029 PMC3313011

[58] C. A. Reinhart‐King , M. Dembo , D. A. Hammer , Biophys. J. 2005, 89, 676. 15849250 10.1529/biophysj.104.054320 PMC1366566

[59] C. R. Foster , J. L. Robson , W. J. Simon , J. Twigg , D. Cruikshank , R. G. Wilson , C. J. Hutchison , Nucleus 2011, 2, 434. 21983087 10.4161/nucl.2.5.17775 PMC3322584

[60] M. Théry , A. Pépin , E. Dressaire , Y. Chen , M. Bornens , Cell Motil. Cytoskeleton 2006, 63, 341. 16550544 10.1002/cm.20126

[61] E. Cukierman , Science 2001, 294, 1708. 11721053 10.1126/science.1064829

[62] K. M. Hakkinen , J. S. Harunaga , A. D. Doyle , K. M. Yamada , Tissue Eng., Part A 2011, 17, 713. 20929283 10.1089/ten.tea.2010.0273 PMC3043991

[63] N. Q. Balaban , U. S. Schwarz , D. Riveline , P. Goichberg , G. Tzur , I. Sabanay , D. Mahalu , S. Safran , A. Bershadsky , L. Addadi , B. Geiger , Nat. Cell Biol. 2001, 3, 466. 11331874 10.1038/35074532

[64] M. Versaevel , T. Grevesse , S. Gabriele , Nat. Commun. 2012, 3, 671. 22334074 10.1038/ncomms1668

[65] S. Sugita , T. Adachi , Y. Ueki , M. Sato , Biophys. J. 2011, 101, 53. 21723814 10.1016/j.bpj.2011.05.046 PMC3127176

[66] K. Katoh , Y. Kano , M. Masuda , H. Onishi , K. Fujiwara , Mol. Biol. Cell 1998, 9, 1919. 9658180 10.1091/mbc.9.7.1919 PMC25437

[67] D. B. Lovett , N. Shekhar , J. A. Nickerson , K. J. Roux , T. P. Lele , Cell. Mol. Bioeng. 2013, 6, 230. 23914256 10.1007/s12195-013-0270-2 PMC3727663

[68] B. Sen , G. Uzer , R. M. Samsonraj , Z. Xie , C. McGrath , M. Styner , A. Dudakovic , A. J. van Wijnen , J. Rubin , Stem Cells 2017, 35, 1624. 28371128 10.1002/stem.2617 PMC5534840

[69] P. M. Davidson , H. Özçelik , V. Hasirci , G. Reiter , K. Anselme , Adv. Mater. 2009, 21, 3586.

[70] F. Badique , D. R. Stamov , P. M. Davidson , M. Veuillet , G. Reiter , J. N. Freund , C. M. Franz , K. Anselme , Biomaterials 2013, 34, 2991. 23357373 10.1016/j.biomaterials.2013.01.018

[71] C. M. Denais , R. M. Gilbert , P. Isermann , A. L. McGregor , M. Te Lindert , B. Weigelin , P. M. Davidson , P. Friedl , K. Wolf , J. Lammerding , Science 2016, 352, 353. 27013428 10.1126/science.aad7297 PMC4833568

[72] J. D. Vargas , E. M. Hatch , D. J. Anderson , M. W. Hetzer , Nucleus 2012, 3, 88. 22567193 10.4161/nucl.18954 PMC3342953

[73] C. Tamiello , M. A. F. Kamps , A. Van den Wijngaard , V. L. R. M. Verstraeten , F. P. T. Baaijens , J. L. V. Broers , C. C. V. Bouten , Nucleus 2013, 4, 61. 23324461 10.4161/nucl.23388 PMC3585029

[74] A. J. Lomakin , C. J. Cattin , J. M. Garcia‐Arcos , I. Y. Zhitnyak , M. K. Driscoll , E. S. Welf , R. J. Petrie , A. M. Lennon‐Duménil , D. J. Müller , Science 2019, 370, eaba2894.

[75] D. M. Jorgens , J. L. Inman , M. Wojcik , C. Robertson , H. Palsdottir , W. T. Tsai , H. Huang , A. Bruni‐Cardoso , C. S. López , M. J. Bissell , K. Xu , M. Auer , J. Cell Sci. 2017, 130, 177. 27505896 10.1242/jcs.190967 PMC5394780

[76] N. Briand , P. Collas , Genome Biol. 2020, 21, 85. 32241294 10.1186/s13059-020-02003-5 PMC7114793

[77] E. M. Balzer , Z. Tong , C. D. Paul , W. C. Hung , K. M. Stroka , A. E. Boggs , S. S. Martin , K. Konstantopoulos , FASEB J. 2012, 26, 4045. 22707566 10.1096/fj.12-211441 PMC3448771

[78] W.‐C. Hung , S.‐H. Chen , C. D. Paul , K. M. Stroka , Y.‐C. Lo , J. T. Yang , K. Konstantopoulos , J. Cell Biol. 2013, 202, 807. 23979717 10.1083/jcb.201302132 PMC3760608

[79] A. Padhi , A. H. Thomson , J. B. Perry , G. N. Davis , R. P. McMillan , S. Loesgen , E. N. Kaweesa , R. Kapania , A. S. Nain , D. A. Brown , Am. J. Physiol.: Cell Physiol. 2020, 318, C476. 31875698 10.1152/ajpcell.00221.2019 PMC7099521

