# Supplementary material for: Sculpting Rupture‐Free Nuclear Shapes in Fibrous Environments
Source: Adv Sci (Weinh). 2022 Jul 21;9(25):2203011. doi: 10.1002/advs.202203011 (PMC9443471; doi:10.1002/advs.202203011)
Supplement: Supplementary file 1 — Supporting Information [file ADVS-9-2203011-s006.pdf]

ADVANCED SCIENCE
Open Access

Supporting Information

for *Adv. Sci.*, DOI 10.1002/advs.202203011

Sculpting Rupture-Free Nuclear Shapes in Fibrous Environments

*Aniket Jana, Avery Tran, Amritpal Gill, Alexander Kiepas, Rakesh K. Kapania, Konstantinos Konstantopoulos and Amrinder S. Nain**

Supporting Information

Sculpting rupture-free nuclear shapes in fibrous environments

Aniket Jana, Avery Tran, Amritpal Gill, Alexander Kiepas ,Rakesh K. Kapania, Konstantinos Konstantopoulos, and Amrinder S. Nain[*]

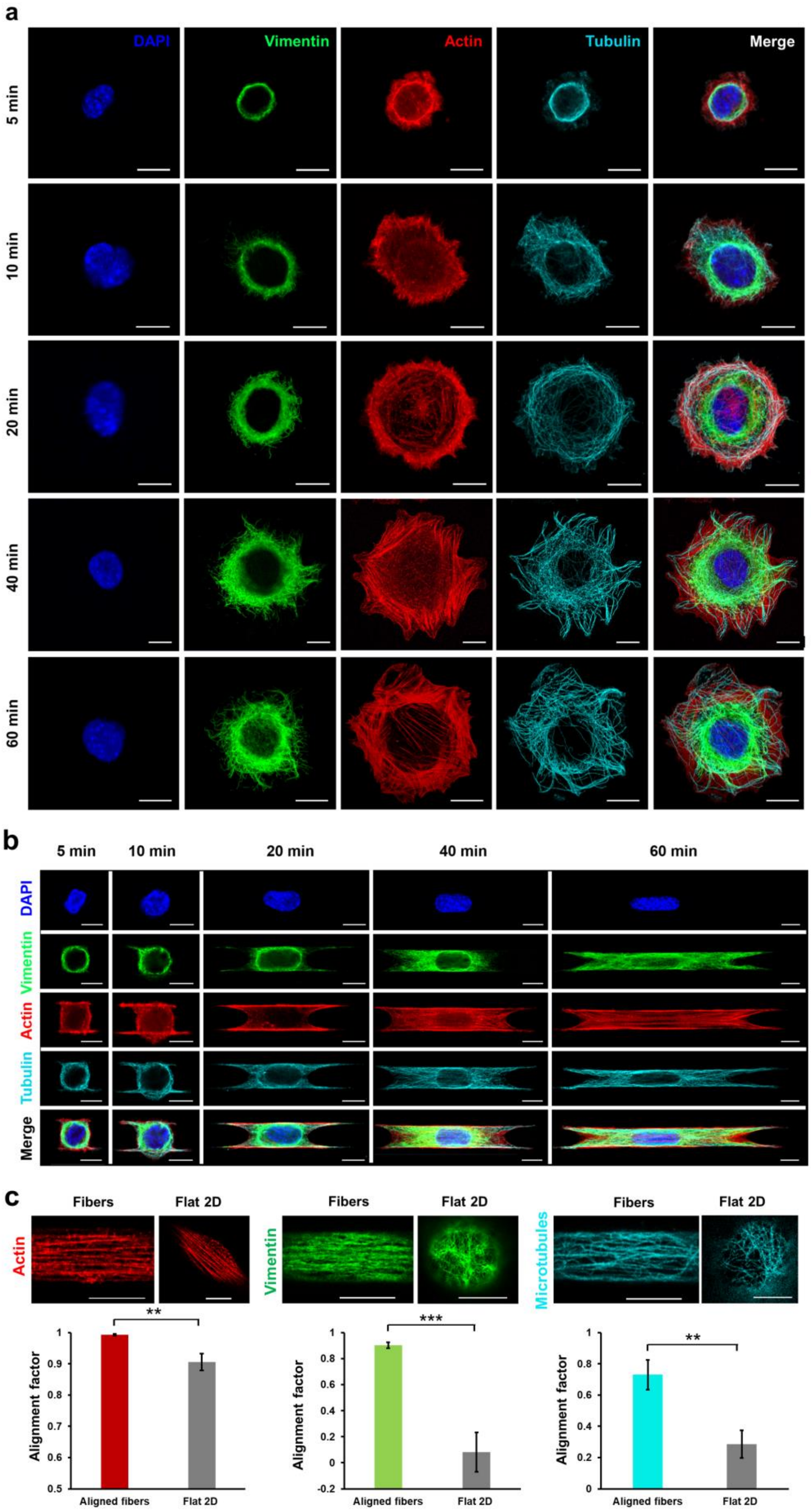
a
DAPI
Vimentin
Actin
Tubulin
Merge
5 min
10 min
20 min
40 min
60 min
b
5 min
10 min
20 min
40 min
60 min
DAPI
Vimentin
Actin
Tubulin
Merge
c
Fibers
Flat 2D
Actin
Vimentin
Microtubules
**
***
**
Alignment factor
Aligned fibers
Flat 2D

Figure S1. Cytoskeletal alignment in suspended fibers a,b) Representative cells showing cytoskeletal organization at different timepoints during spreading on flat glass and suspended fiber doublets (fiber diameter: 350 nm) respectively, cells are stained for actin (red), vimentin intermediate filaments (green), microtubules (cyan) and nuclei (blue), cells on fibers show entry of microtubules and intermediate filaments into the protrusions as spreading progresses, scale bars represent 10 µm c) Comparison of the alignment of the cytoskeletal elements in the perinuclear region, between flat glass and suspended fibers (fiber diameter: 350 nm). Alignment factor is defined as cos(2α) where α is the orientation of the actin stress fiber or individual pores within the microtubule or intermediate filament meshwork, with respect to the primary cell orientation, Alignment factor ~ 1 for perfect alignment and ~ 0 for random alignment, scale bars represent 10 µm, n=5-6 cells for aligned fibers and Flat 2D respectively.

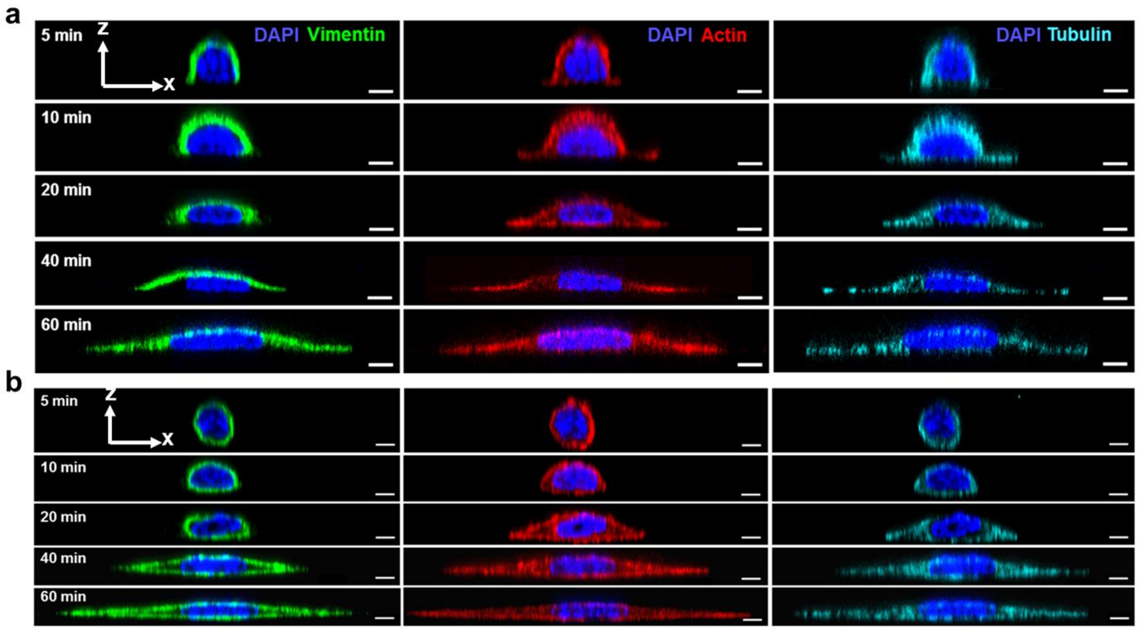

Figure S2. Spatiotemporal localization of cytoskeletons a) Confocal side views (xz) showing 'Capping' localization of the cytoskeletal elements at various timepoints during cell spreading on flat glass b) 'Caging' localization observed in suspended fibers (fiber diameter: 350 nm), actin (red), vimentin intermediate filaments (green), microtubules (cyan) and nuclei (blue), all scale bars represent 5 µm

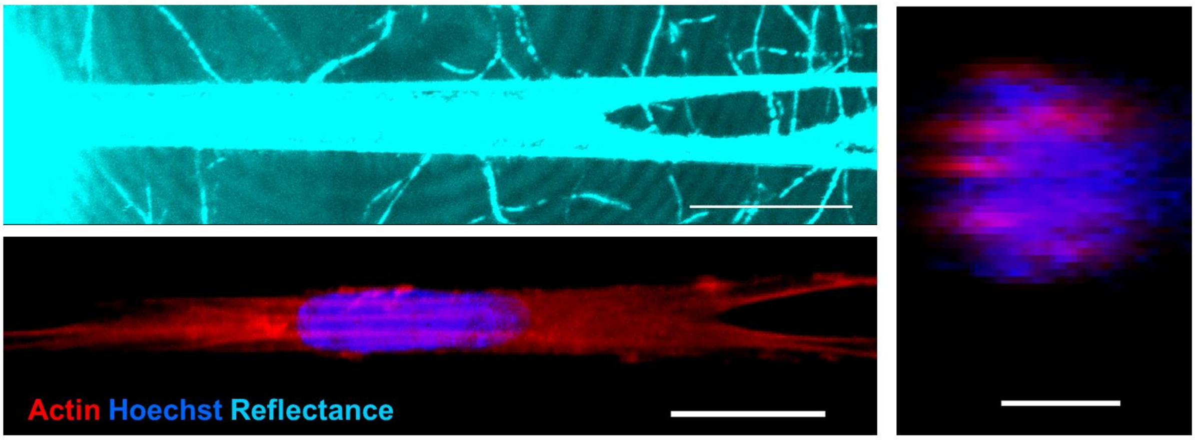

Figure S3. Cytoskeletal caging of the nucleus in cells attached to nanofiber networks embedded in 3D collagen gels

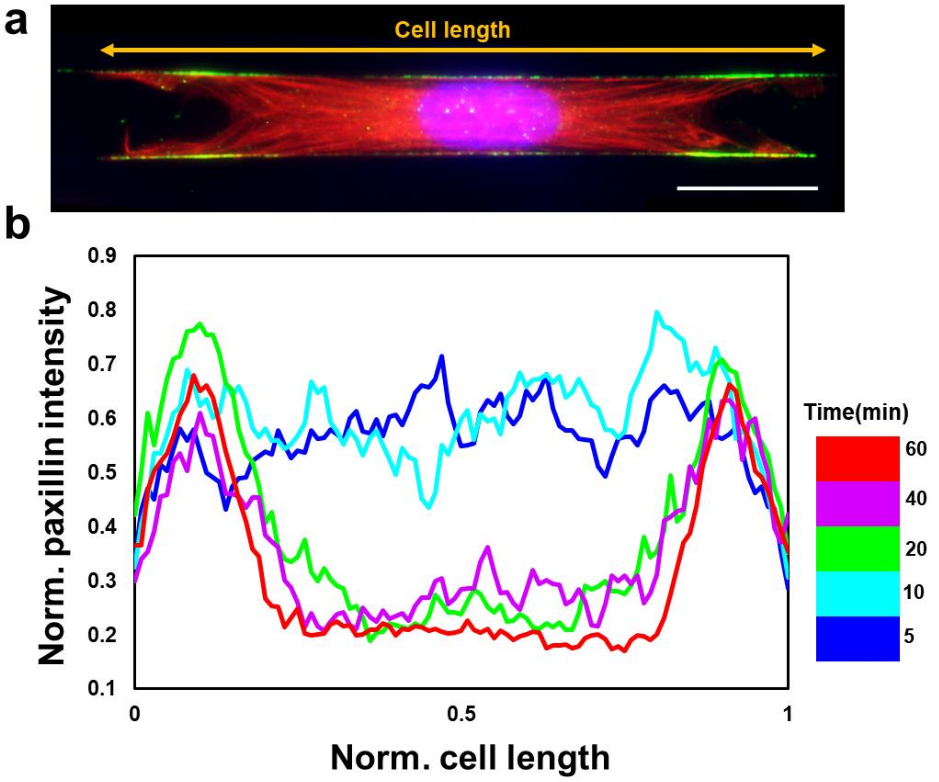

Figure S4. Evolution of focal adhesion organization over the course of cell spreading a) Representative spread cell showing focal adhesion clustering at cell poles, scale bars represent 20 µm, b), Average profiles (n=9-12 for each timepoint) are shown, Paxillin intensity is normalized with respect to the peak intensity of each profile, Cell length normalization was performed with respect to the entire length of the cell as shown in part a, In the spread state, two intensity peaks demonstrate the focal adhesion clustering, Data is shown for 350 nm diameter category

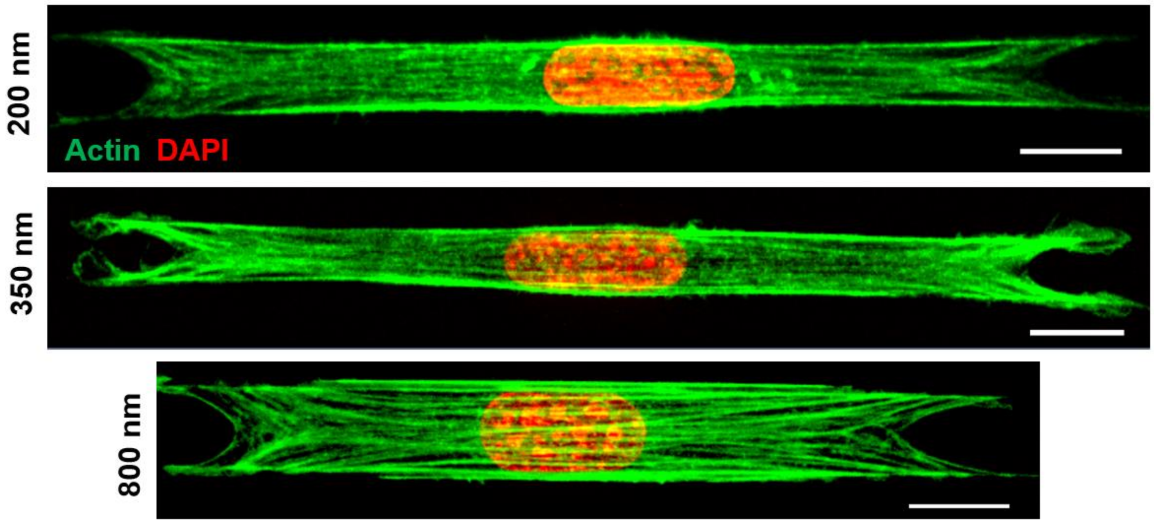

Figure S5. Stress fiber organization in spread cells, Maximum intensity projections of representative cells demonstrating stress fibers emerge from focal adhesion clusters on either side and converge to become almost parallel to the cell orientation, in the perinuclear region. Actin and DAPI are pseudo-colored in green and red for better visualization. Scale bars represent 10 μm.

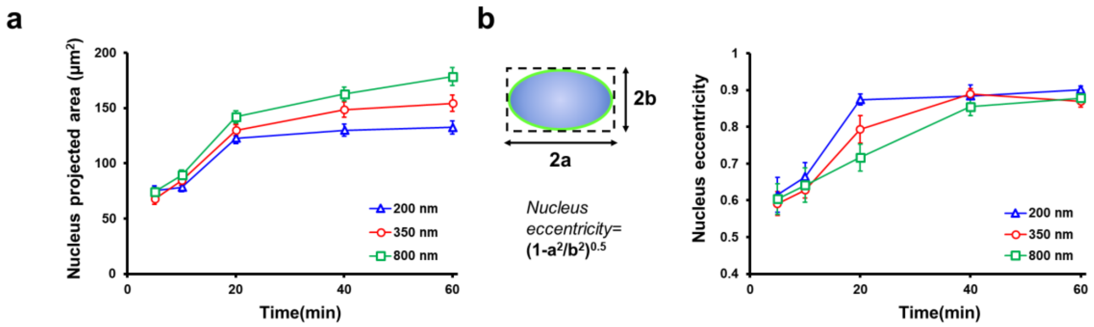

Figure S6. Nucleus geometry during cell spreading a,b) Evolution of nucleus projected area (xy) and eccentricity as function of spreading time and shown for the different diameter fiber doublets, respectively, n=17-34 per timepoint for each diameter category

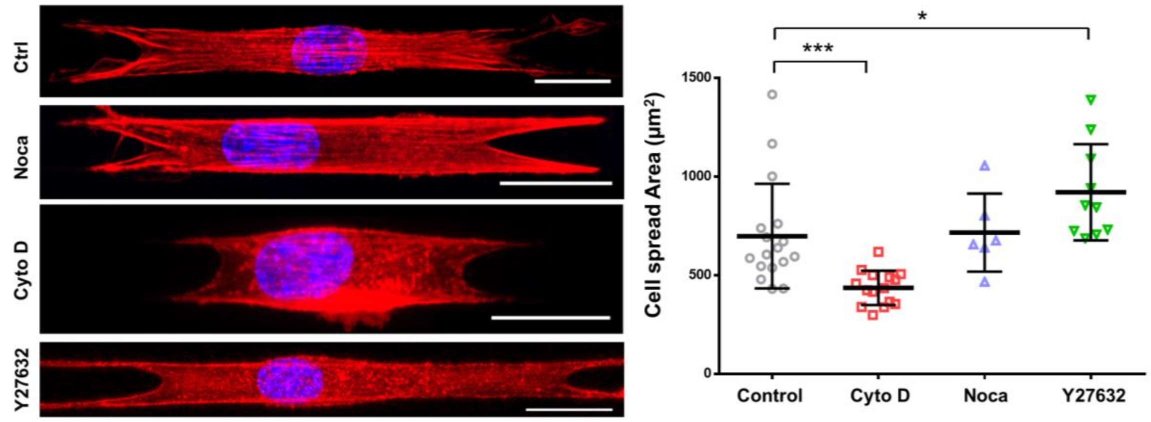

Figure S7. Effect of cytoskeletal inhibition on cell spreading, Representative images showing control (no treatment), cytochalasin D, nocodazole and Y27632 treated cells, fiber diameter: 350 nm, scale bars represent 20 μm. Comparison of the cell spread area under different drug conditions, n=17,15,6 and 10.

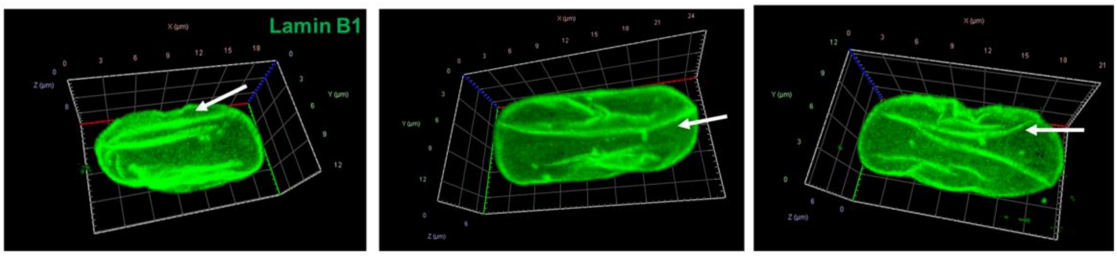

Figure S8. Morphological alterations in Lamin A/C KD cells a) 3D isometric views showing wrinkled morphologies of Lamin A/C KD cells, white arrows indicate wrinkles, Lamin B1 is shown in green

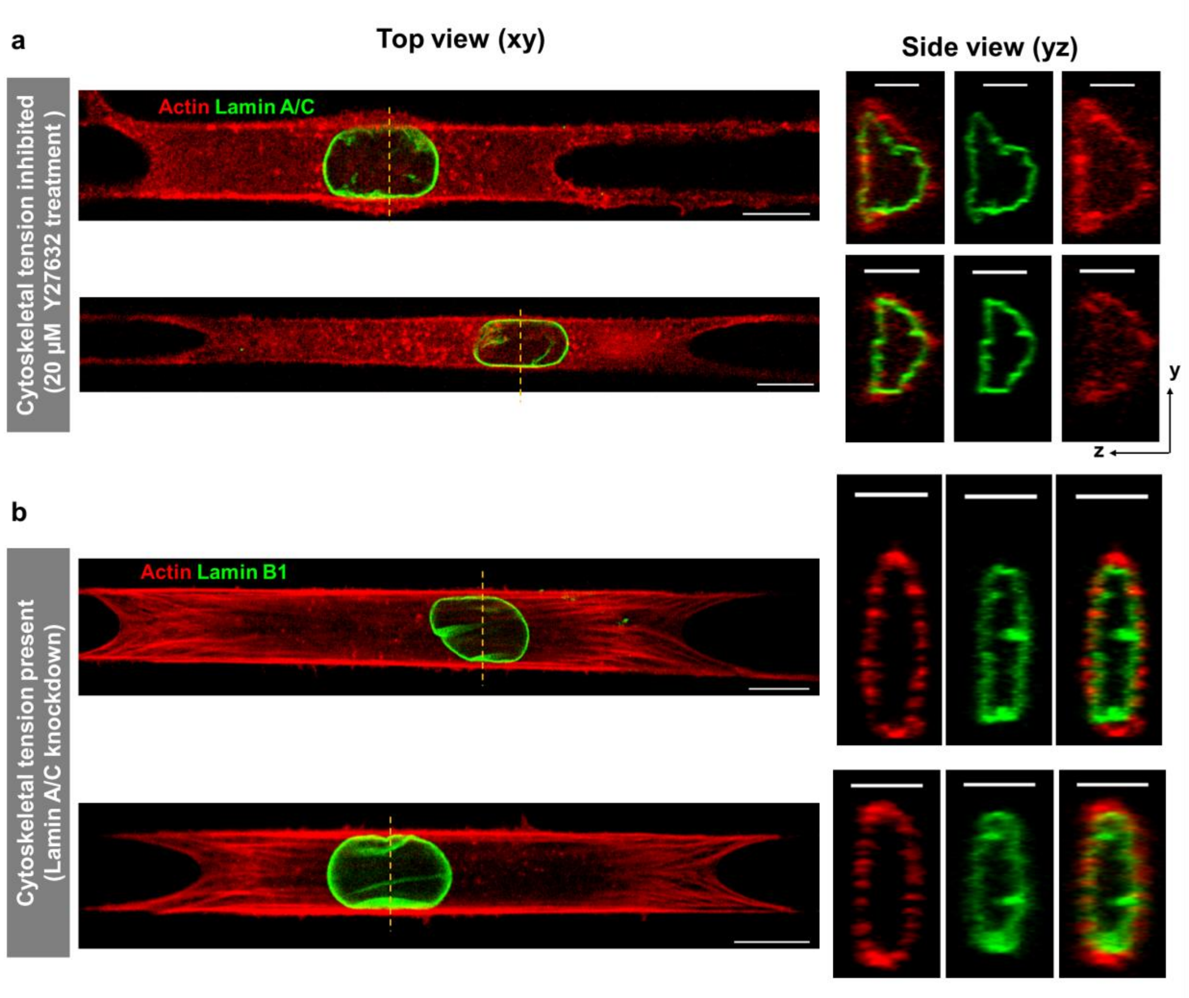

Figure S9. Influence of cytoskeletal caging towards nuclear shape and lamina organization, a) Representative C2C12 cells with inhibited cytoskeletal tension through 20 μM Y27632 treatment, and stained for actin and Lamin A/C, Cross-sectional views demonstrate significantly asymmetric nuclear shapes. b) Representative C2C12 Lamin A/C KD cells stained for actin and Lamin B1, cross-sectional views demonstrate cytoskeletal caging of the nucleus is still present, Scale bars (top panel 10 μm and bottom side-view panels are 5 μm).

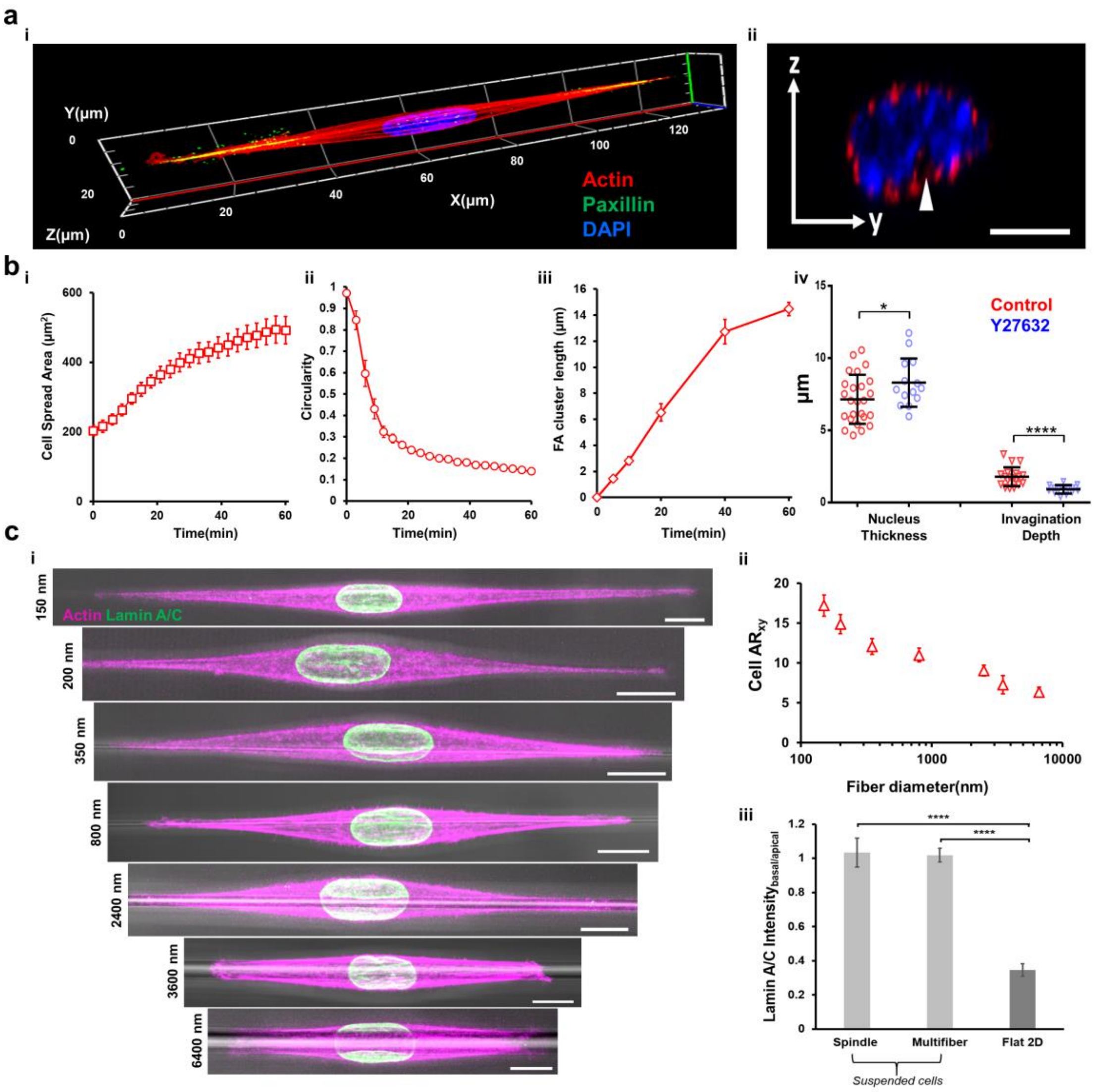

Figure S10. Cell and nucleus shapes in spindle cells: a) i) 3D isometric view of spindle cell stained for actin (red), paxillin (green, shown with yellow arrows) and nucleus (blue) ii) Representative confocal side views (yz) showing stress-fiber mediated caging of nucleus in spindle shaped cells, scale bars represent 5 µm b) i.ii) Temporal evolution of cell spread area and circularity for spindle cells, n=15 cells, fiber diameter: 350 nm, iii) Focal adhesion cluster (FAC) length evolution during cell spreading, n=34.47,32,17 and 34 for 5, 10,20,40 and 60 min respectively, iv) Effect of cell contractility inhibition on nucleus thickness and invagination size in spindle cells, (fiber diameter: 350 nm) c) i) Representative images showing variation of cell/nucleus shape as the fiber diameter is varied from 150-6600 nm. Actin (magenta) and lamin A/C (green) are overlaid with the brightfield channel to show underlying fibers, scale bars represent 10 µm ii) Cell aspect ratio as a function of fiber diameter, n=14,10,10,11,10,6 and 6 for 150,200,350,800,2500,3500 and 6600 nm respectively iii) Localization of lamin A/C at the basal (contact points with fiber) and apical surfaces for spindle (1-fiber) and multifiber configurations, Flat 2D is included for comparisons (n=5-10 cells).

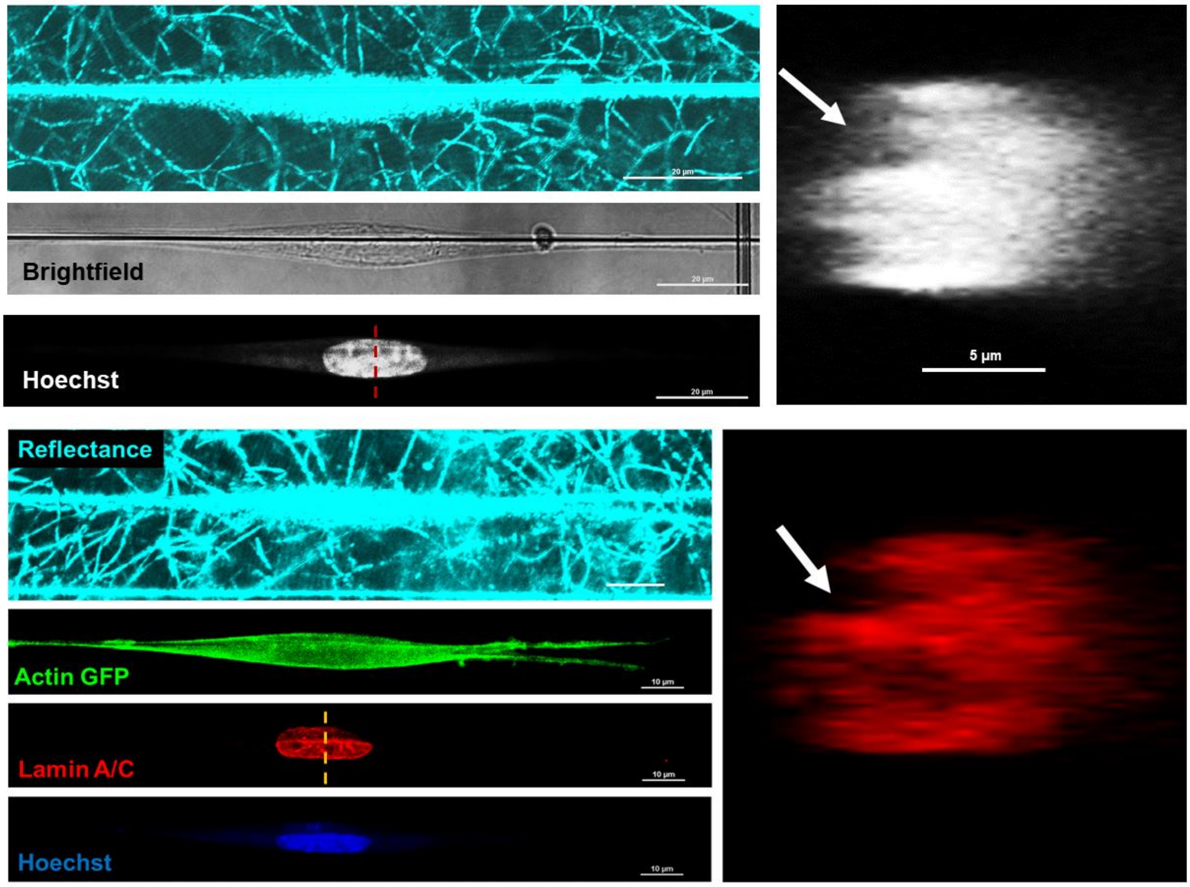

Figure S11. Identification of nuclear invaginations in cells inside 3D adhesive spaces , Representative elongated cells attached to nanofiber networks inside 3D collagen gels, Individual collagen fibrils visualized through confocal reflectance microscopy

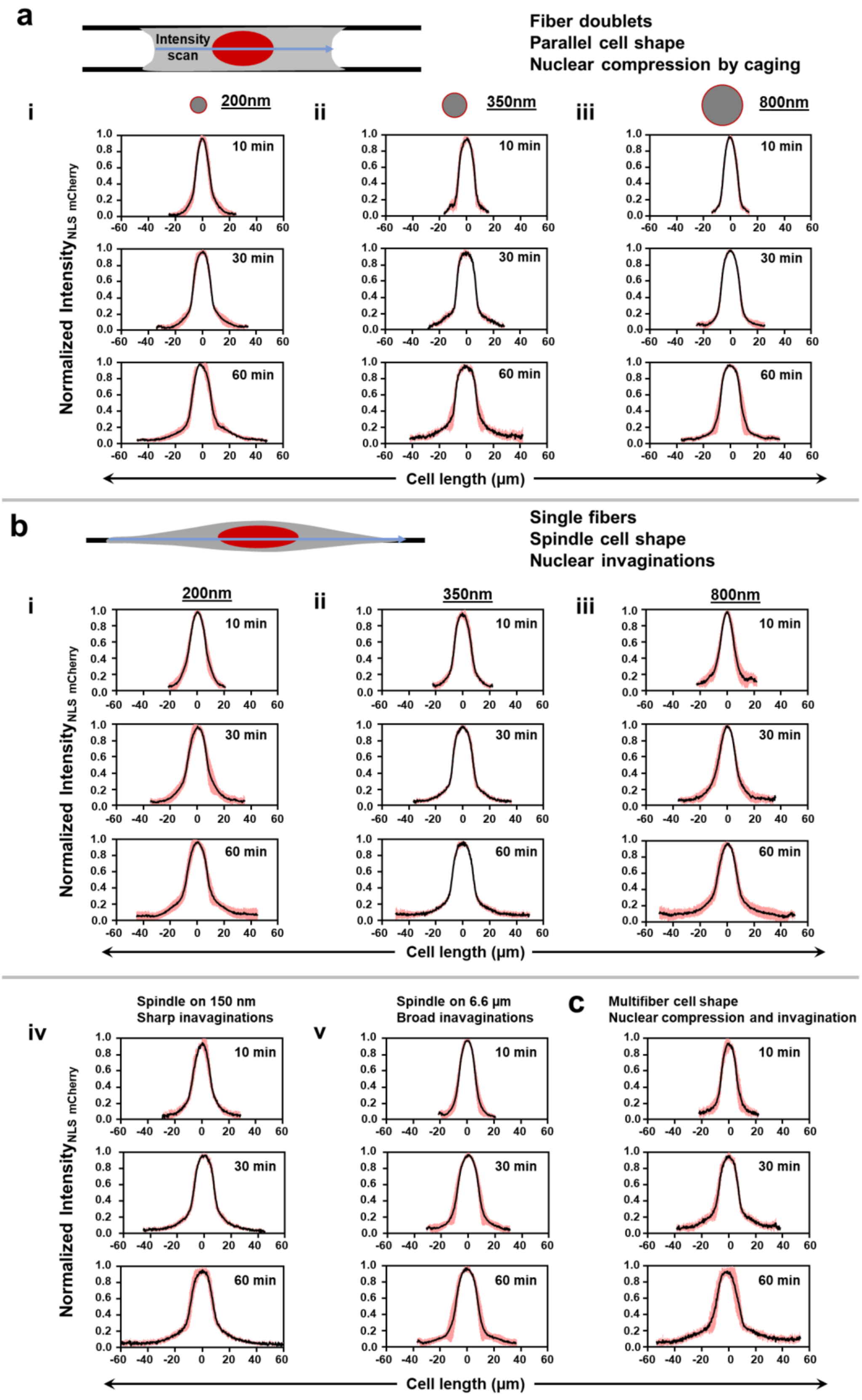

Figure S12. NLS localization in aligned fibers a) i,ii,iii) NLS intensity profiles, along cell length at different spreading timepoints (~ 10,30,60 min) corresponding to fiber doublets with 200 nm , 350 nm and 800 nm respectively, Schematic illustrates the direction of intensity scans b) i,ii,iii, iv and v) Spatiotemporal NLS localization for spindle shaped cells corresponding to fiber diameters: 200, 350 ,800, 150 and 6600 nm respectively c) Intensity analysis for multifiber (D: 350 nm) cells, n=10 cells for each timepoint and substrate category. Error bars represent standard deviation.

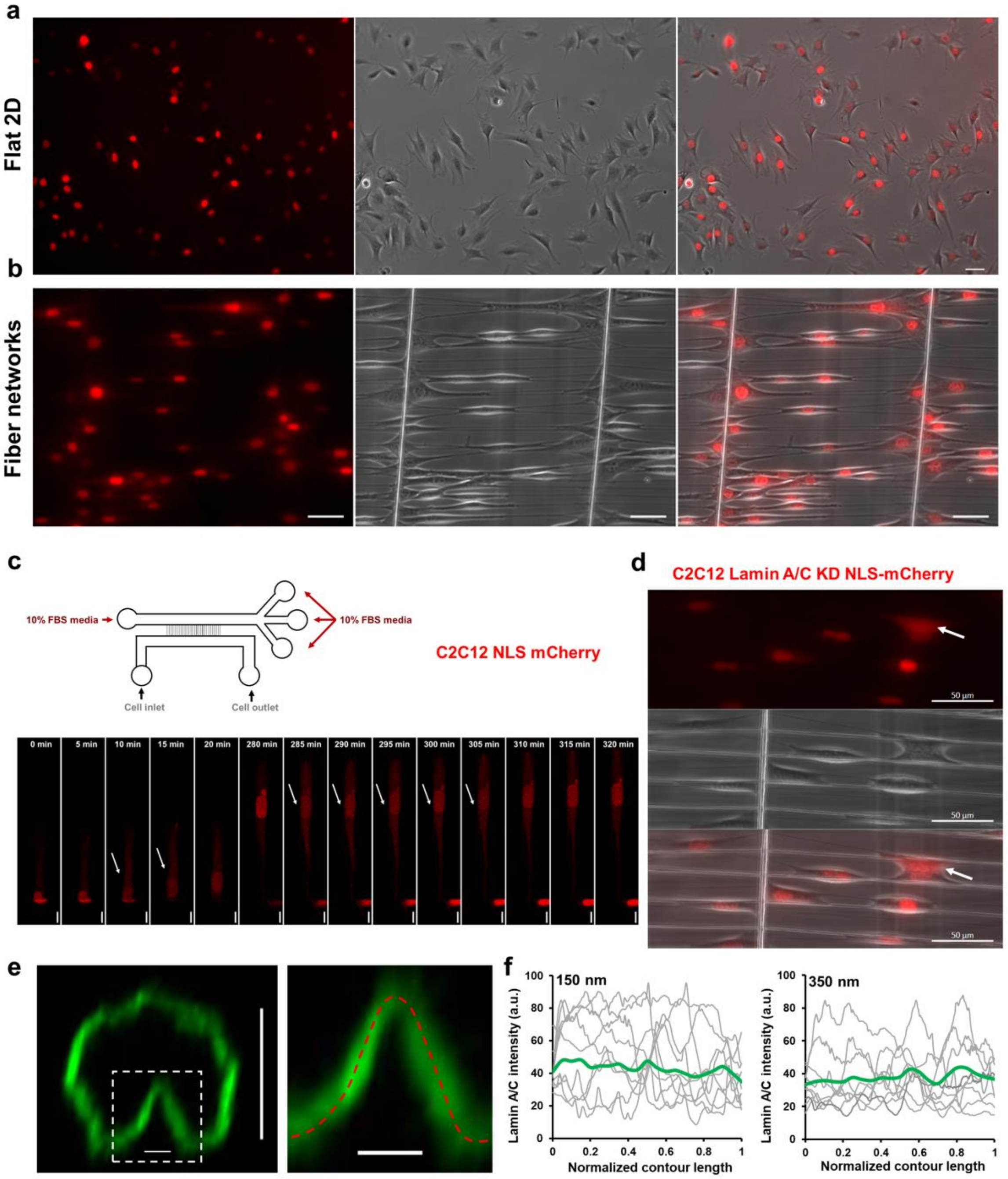

Figure S13. a,b) Large field of view showing several C2C12 WT cells expressing NLS mCherry, adhered to Flat 2D and fiber networks, scale bars represent 50 µm, c) Schematic for the microfluidic channel intended for nuclear constriction, Representative timelapse images of C2C12 NLS mCherry cell moving through a microchannel showing rupture events (marked by white arrows), scale bars represent 5 µm, d) Representative image showing nuclear rupture (marked by white arrow) in C2C12 Lamin A/C KD cells e) Representative cross-sectional view of nuclear lamina (Lamin A/C stained in green) with inset showing region of invagination, line scan is performed along the entire contour length of the invagination (marked by the red -dotted line) , scale bars represent 5 µm and 1 µm respectively, f) Individual intensity scan profiles (shown in gray) and the averages profile (shown in green) corresponding to small diameter nanofibers (150 nm, 350 nm). Length is normalized with respect to the net contour length corresponding to each profile.

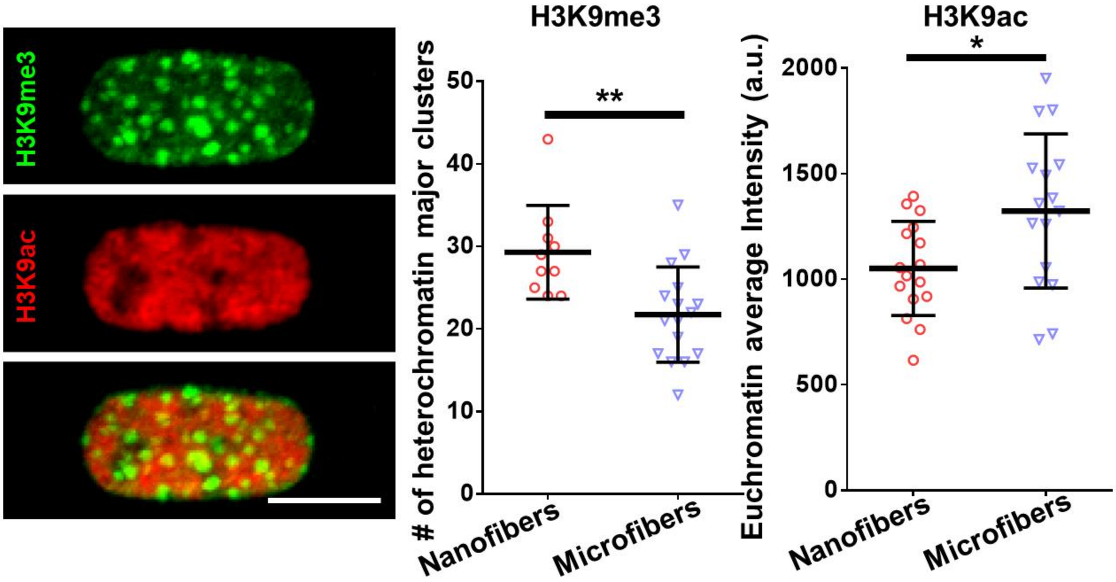

Figure S14: Influence on invagination shape on chromatin characterestics , Representative image (maximum intensity projection) showing nuclei stained for euchromatin marker (H3k9ac, shown in red) and heterochromatin marker (H3K9me3, shown in green). A number of major heterochromatin foci are measured at the spread state (1 hr) and compared between nanofibers and microfibers. Immunofluorescence intensity of H3K9ac was used for relative comparison of euchromatin content. Scale bars represent 10 µm. Student's t-test used, *, ** represents $p<0.05$ and 0.01 respectively.

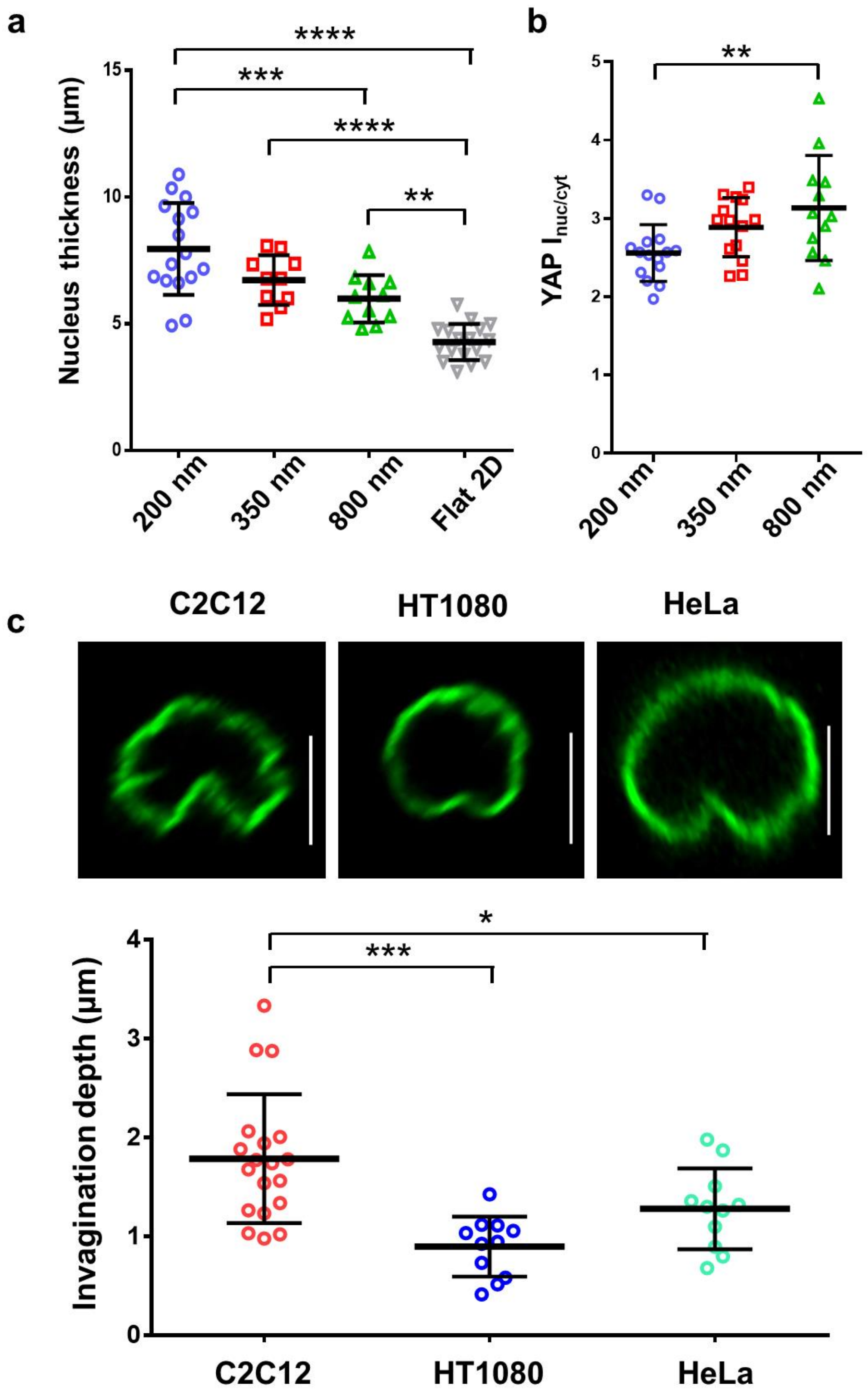

Figure S15. Nucleus shape regulation mode is independent of cell line: a) Fiber diameter dependence of nucleus thickness in HT1080 fibrosarcoma cells b) Enhanced nuclear translocation of YAP for HT1080 cells in 800 nm networks. c) Nuclear invaginations are observed in spindle cells for all cell types tested (C2C12, HT1080 and HeLa), comparison of the invagination depths in the different cell types for 350 nm diameter single fibers, scale bars represent 5 µm.

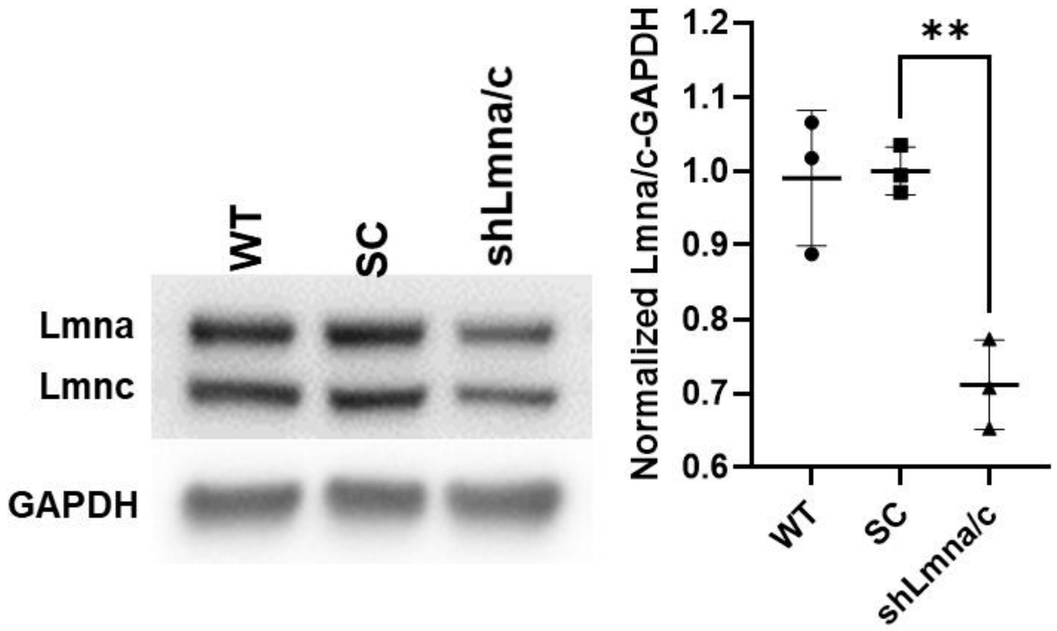

Figure S16: Western blot showing partial knockdown of Lamin A/C in C2C12 cells. SC stands for scramble with null expression.
